# Clinical applications of fundus autofluorescence in retinal disease

**DOI:** 10.1186/s40942-016-0035-x

**Published:** 2016-04-08

**Authors:** Madeline Yung, Michael A. Klufas, David Sarraf

**Affiliations:** 1 Stein Eye Institute, David Geffen School of Medicine at University of California, Los Angeles, CA 90095 USA; 2 Greater Los Angeles VA Healthcare Center, Los Angeles, CA 90024 USA

**Keywords:** Fundus autofluorescence, Retina, Imaging, Lipofuscin, Age related macular degeneration, Central serous retinopathy, Macular dystrophy, Retinitis pigmentosa, White dot syndrome, Hydroxychloroquine

## Abstract

Fundus autofluorescence (FAF) is a non-invasive retinal imaging modality used in clinical practice to provide a density map of lipofuscin, the predominant ocular fluorophore, in the retinal pigment epithelium. Multiple commercially available imaging systems, including the fundus camera, the confocal scanning laser ophthalmoscope, and the ultra-widefield imaging device, are available to the clinician. Each offers unique advantages for evaluating various retinal diseases. The clinical applications of FAF continue to expand. It is now an essential tool for evaluating age related macular degeneration, macular dystrophies, retinitis pigmentosa, white dot syndromes, retinal drug toxicities, and various other retinal disorders. FAF may detect abnormalities beyond those detected on funduscopic exam, fluorescein angiography, or optical coherence tomography, and can be used to elucidate disease pathogenesis, form genotype-phenotype correlations, diagnose and monitor disease, and evaluate novel therapies. Given its ease of use, non-invasive nature, and value in characterizing retinal disease, FAF enjoys increasing clinical relevance. This review summarizes common ocular fluorophores, imaging modalities, and FAF findings for a wide spectrum of retinal disorders.

## Background

Fundus autofluorescence (FAF) is a non-invasive imaging technique that detects fluorophores, naturally occurring molecules that absorb and emit light of specified wavelengths [1]. To produce autofluorescence, a fluorophore absorbs a photon of the excitation wavelength, which elevates an electron to an excited, high energy state. The electron dissipates some energy through molecular collisions, then emits a quantum of light at a lower energy and longer wavelength as it transitions back to ground state. Classically, FAF utilizes blue-light excitation, then collects emissions within a preset spectra to form a brightness map reflecting the distribution of lipofuscin, a dominant fluorophore located in the RPE. FAF may use other excitation wavelengths to detect additional fluorophores, such as melanin with near-infrared autofluorescence.

First described by Delori in the 1980s, FAF has since expanded in both scope and practice [1]. Given the unique findings not identified with funduscopic examination, fundus photography, or fluorescein angiography, FAF is useful in the evaluation of a diverse spectrum of diseases involving the retina and RPE, including degenerative, dystrophic, inflammatory, infectious, neoplastic, and toxic etiologies. This review summarizes the known ocular fluorophores, various imaging modalities, and broad clinical applications of FAF.

## Ocular fluorophores: lipofuscin, A2E, and other ocular fluorophores

### Lipofuscin

**Fig. 1:**
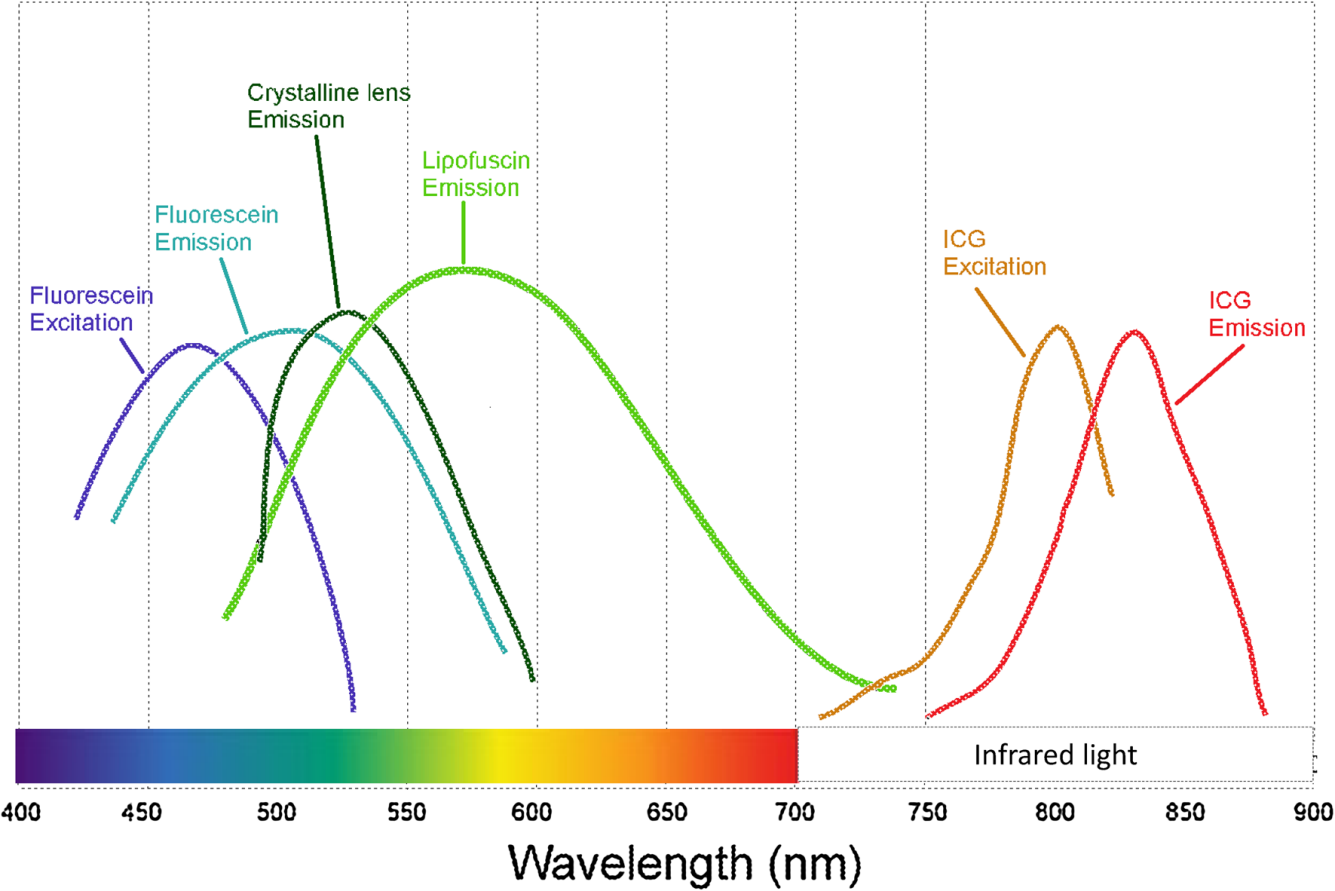
Excitation and emission spectra of common retinal imaging modalities. Fluorescein angiography and fundus autofluorescence operate in the blue–green spectra of visible light, while indocyanine green angiography (ICG) utilizes the infrared spectra. The crystalline lens, a common source of interference, has an emission peak similar to that of lipofuscin. With kind permission from Springer Science + Business Media: Atlas of Fundus Autofluorescence Imaging, Chapter 5: Autofluorescence Imaging with the Fundus Camera, 2007, pp. 49–54, Spaide, Fig. 5.1

Located in the RPE, lipofuscin is a dominant macular fluorophore that absorbs blue light with a peak excitation wavelength of 470 nm and emits yellow–green light at a peak wavelength of 600–610 nm (Fig. 1) [2]. Lipofuscin is a heterogeneous mixture and derives its autofluorescent properties from bisretinoid compounds, which are metabolic byproducts of vitamin A and the visual cycle.

Bisretinoids are initially formed in photoreceptor outer segments and then deposited in the RPE as lipofuscin, accumulating in RPE lysosomes with age [1]. Lipofuscin also increases in degenerative disorders, including age-related macular degeneration (AMD), and macular dystrophies such as Best and Stargardt disease [3]. The distribution of lipofuscin, and consequently the distribution of autofluorescence, is greatest in the posterior pole but limited in the fovea, and decreases toward the periphery [1, 4].

### A2E

*N*-Retinyl-*N*-retinylidene ethanolamine (A2E) is the first and best characterized component of lipofuscin, with excitation at 430–450 nm and maximum emission at 560–575 nm [5]. A pyridium bisretinoid, A2E is enzymatically indigestible, accumulates in RPE lysosomes, exerts multiple toxic effects on RPE cells in vitro, and has been implicated in multiple degenerative retinal diseases. When irradiated by blue-light, A2E undergoes photo-oxidation and generates reactive oxygen species [6–10]. In addition, A2E has been shown to interfere with cholesterol metabolism, destabilize cell membranes, damage DNA, and trigger apoptosis [11–13]. However, other studies suggest that A2E may in fact protect the retina from photo-oxidative stress. A2E generates singlet oxygen species much less effectively than its precursor all-trans-retinal, and conversion to A2E may protect the retina from the toxic effects of all-trans-retinal. A2E accumulation may be merely a marker for aberrant visual cycle activity, rather than the source of retinal damage [14, 15]. In addition, A2E is distributed peripherally in human eyes, rather than centrally, like lipofuscin, suggesting that A2E may not be the dominant fluorophore responsible for increased macular FAF over time [16–18]. Given these mixed results, the role of A2E in retinal disease appears complex and requires further study to fully elucidate its function.

### Other ocular fluorophores

Other clinically significant fundus fluorophores include vitelliform lesions and optic disc drusen. Vitelliform lesions refer to the clinical finding of round, yellow, retinal lesions reminiscent of an egg yolk. While lipofuscin is located within RPE lysosomes, vitelliform lesions consist of extracellular fluorophores—shed outer segment debris in the subretinal extracellular space, which accumulate due to RPE dysfunction and loss of apposition between photoreceptor tips and the RPE [19–21].

Optic disc drusen are deposits of extracellular mitochondria in a filamentous protein matrix and if superficial, may produce increased FAF [22]. These lesions may be associated with visual field defects, optic nerve dysfunction, and various vitreoretinal conditions including retinitis pigmentosa, Alagille syndrome, and pseudoxanthoma elasticum [23].

Structures anterior to the retina, including the cornea and lens, naturally emit autofluorescence and can cause interference, decreasing image resolution in FAF systems. The cornea has an excitation peak at 365–480 nm and an emission peak at 620 nm [24], while the lens has an excitation peak at 420–430 nm and an emission peak at 520 nm [25]. Diseases involving these structures can further impact FAF findings. For example, corneal autofluorescence increases in patients with diabetes and is thought to result from the accumulation of advanced glycation products [26, 27]. Cataracts increase light absorption and scatter by the lens, leading to poor contrast autofluorescence images that improves with cataract extraction and intraocular lens placement [28, 29].

In order to minimize interference from the lens and cornea, fundus cameras have adopted barrier filters with red-shifted wavelengths. Confocal scanning laser ophthalmoscopes (cSLO) utilize confocal optics in the form of a spatial pinhole that collects light from a single optical plane at the level of the fundus while eliminating out-of-focus light [30–32].

### Melanin

Melanin is an ocular pigment located in the both RPE cells and in uveal melanocytes. In the fundus, melanin is distributed primarily in the fovea, macula, and periphery [4]. Within RPE cells, melanin granules are located apically and lipofuscin basolaterally [33, 34]. In contrast to lipofuscin, melanin has a peak excitation at a longer wavelength of 787 nm and is the primary fluorophore in near-infrared autofluorescence [35]. On conventional FAF, melanin absorbs the short-wavelength excitation beam, decreasing the overall autofluorescent signal [5].

Melanin protects the retina from light-induced damage in several ways [36]. Melanin located in anterior segment structures such as the iris absorb and block visible light and UV radiation, shielding the retina from excessive light energy. In addition, RPE melanin acts as an antioxidant, protecting against free radicals, redox-reactive heavy metals, photo-oxidation, and lipofuscin accumulation [37–41], although this effect decreases with age and melanin may even gain pro-oxidant properties over time [40]. Individuals with lightly pigmented irises [42–44] and decreased RPE melanin content [45] demonstrate a higher incidence and severity of age-related macular degeneration.

### Rhodopsin

**Fig. 2:**
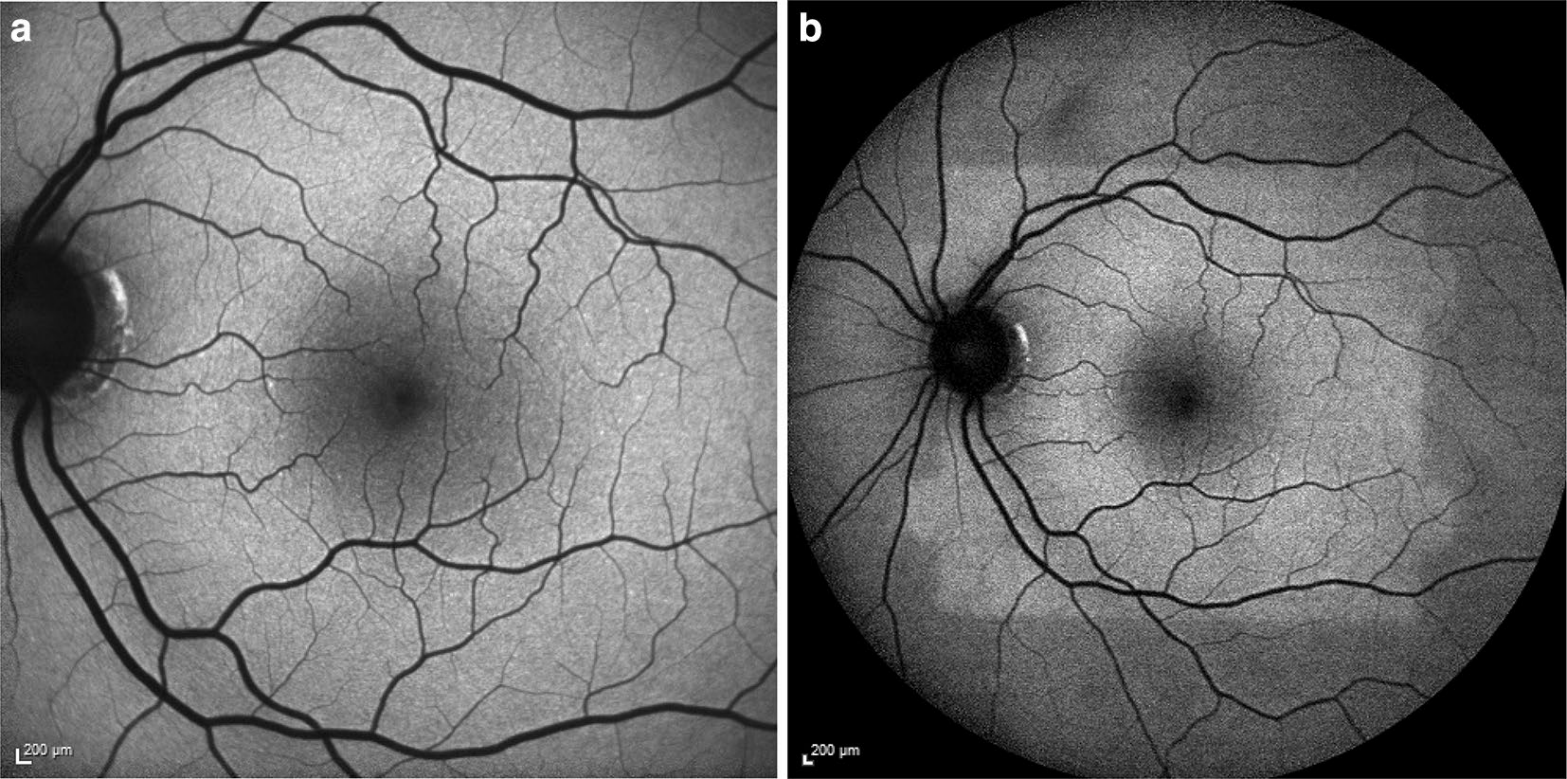
Bleaching effect of rhodopsin. Immediately after obtaining a 30 degree FAF (**a**), the 50 degree FAF (**b**) shows a discrete area of relative hyper-autofluorescence corresponding to an area of bleached photoreceptors resulting from the first image acquisition

Rhodopsin is a visual pigment concentrated in rod photoreceptor outer segments that absorbs the excitation beam and decreases autofluorescence [46]. However, with continued exposure to light, rhodopsin undergoes photo-isomerization and loses its absorptive capabilities, resulting in a progressive increase in autofluorescent signal [47]. Termed the bleaching effect, rhodopsin absorption and photo-isomerization is seen with short-wavelength, but not near-infrared, excitation beams. Compared to dark-adapted eyes in which rhodopsin is maximally active, FAF can increase over 30 % after exposure to light that strongly bleaches rhodopsin (Fig. 2) [48]. Changes in optical density due to the bleaching effect are decreased in retinal dystrophies involving photoreceptor dysfunction, including cone-rod dystrophy, Stargardt disease, and choroideremia [49].

## Commonly used fundus autofluorescence (FAF) imaging systems

Commercially available FAF systems include fundus cameras, confocal scanning laser ophthalmoscopes (cSLO), and ultra-widefield technologies (Table 1). Limitations of FAF include a low signal strength (two orders of magnitude less than the peak signal of fluorescein angiography) and autofluorescence artifact from anterior segment structures. In addition, the blue-light excitation beam may cause patient discomfort and be potentially toxic to the retina, although there have been no formal studies demonstrating the adverse effects of FAF. The various imaging modalities have utilized different strategies to address these shortcomings.

**Table 1:** Fundus autofluorescence imaging modalities. Excitation wavelengths, barrier filters, fields of view, advantages, and disadvantages of commercially available FAF systems. Although some systems use multiple wavelengths, only the FAF excitation wavelength is provided

Imaging modality	Fundus autofluorescence imaging systems	Excitation wavelength	Barrier filter (nm)	Field of view	Advantages	Disadvantages
Fundus camera					Better for visualizing exudative retinal disease, red-shifted wavelengths decrease absorption by macular pigments and reduce lens interference, can be used with FA, color imaging, decreased motion artifact, more comfortable for patient	No real-time averaging, poor contrast, capture more reflected and scattered light, prone to pseudo-autofluorescence
	Topcon TRC-50DX	535–585 nm	615–715	20, 35, 50	Non-mydriatic, also offers FA, ICG	
	Zeiss Visucam 224/524	510–580 nm	650–735	30, 45	Non-mydriatic. Visucam 524 with FA and optional ICG	
	Canon CR-2 plus AF (non mydriatic)	530–580	640	35, 45	Non-mydriatic, also offers cobalt setting	
Confocal scanning laser ophthalmoscope (cSLO)					Confocal optics reduces interference from the lens, real-time averaging, high contrast, high resolution, decreased scattered light	Excitation beam is absorbed by macular pigments, cannot be preceded by fluorescein angiography, fixation loss, monochromatic, patient discomfort
	Heidelberg retinal angiograph (HRA 2)	488 nm	500	20, 30, 55	No longer commercially available	
	Heidelberg spectralis	488 nm	500	20, 30, 55	Also offers red-free, FA, ICG, simultaneous FA/ICG, infrared reflectance, multicolor imaging, dual wavelength technology can calculate macular pigment density, spectral domain OCT	
	Zeiss prototype SM 30 4024 (ZcSLO)	488 nm	521	20, 40	No longer commercially available	
	Rodenstock (RcSLO)	488 nm	515	20, 40	No longer commercially available	
	Nidek F-10	490 nm	510	40, 60	Also offers multicolor imaging, retro-mode, FA, ICG	
Widefield cSLOs					Detects peripheral findings, non-mydriatic, brief image acquisition time, can be used with FA	Disadvantages vary by system and lens
	Optos ultra-widefield	532 nm, 633 nm	540	200	Decreased absorption by macular pigments, also offers color fundus, red-free, FA, ICG	No real-time averaging, poor contrast, distortion of peripheral retina, view limited in superior and inferior quadrants, lid/lash artifact
	Staurenghi lens	N/A	N/A	150	Lens attaches to cSLO	Requires placement of contact lens
	Heidelberg ultra-widefield lens	N/A	N/A	105	Lens attaches to HRA or Spectralis. High contrast, non-distorted images, no lid/lash artifact, can be used with fluorescein angiography	Smaller field of view, view limited in nasal and temporal quadrants

*cSLO* confocal scanning laser ophthalmoscope, *ICG* indocyanine green angiography, *FA* fluorescein angiography, *OCT* optical coherence tomography

### Fundus camera

**Fig. 3:**
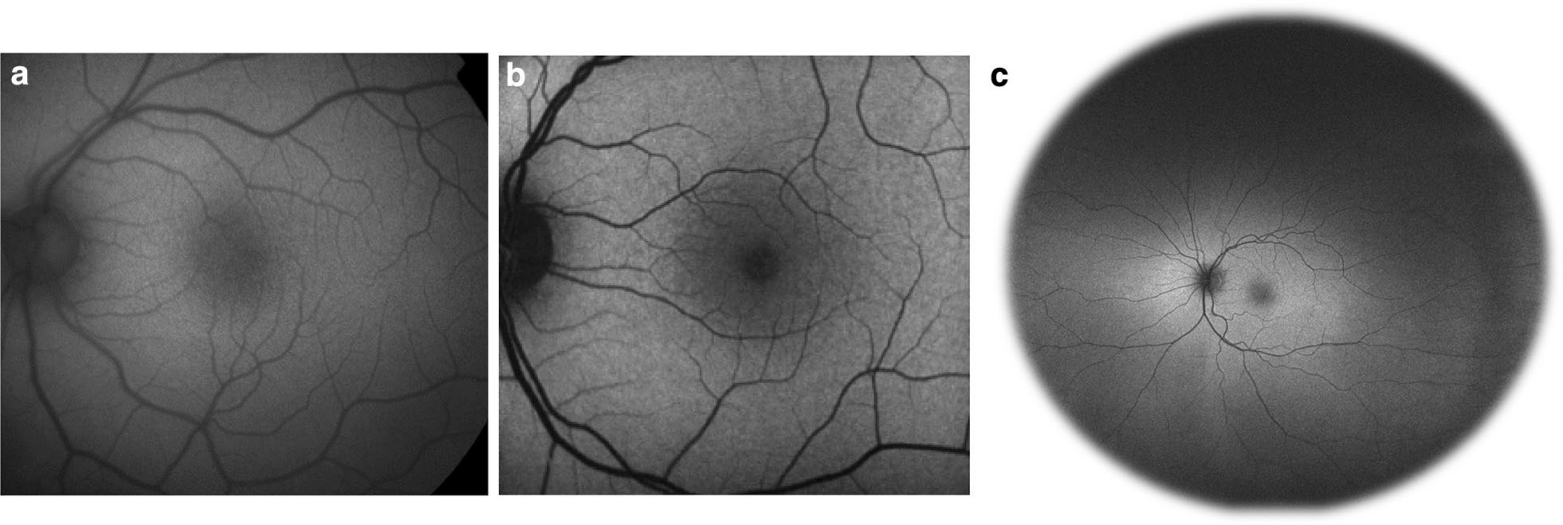
Comparison of common imaging systems available for fundus autofluorescence. Images of normal, healthy retina were obtained using the fundus camera with a Spaide filter (**a**), confocal cSLO (**b**), and Optos ultra-widefield systems (**c**)

The fundus camera is a digital system that captures autofluorescence using a single flash of light (Fig. 3a) [50]. Commercially available models are produced by Topcon^®^, Zeiss^®^, and Canon^®^ (Table 1). To reduce autofluorescence of the lens and cornea, Spaide introduced the ‘modified Topcon’ filter set with red-shifted wavelengths, with an excitation spectra of 535–585 and a 615–715 nm emission barrier filter [51].

The use of the red-shifted wavelengths by fundus cameras decreases absorption by macular pigments, allowing detection of subtle perifoveal RPE changes [50]. This excitation spectra also decreases absorption by the crystalline lens, preserving image quality in cases of cataract. In fact, fundus cameras demonstrate reduced image degradation and higher rates of successful image acquisition in patients with cataracts compared to cSLOs [52, 53]. Fundus cameras also provide better detection of exudative retinal disease such as choroidal neovascularization or central serous chorioretinopathy compared to cSLOs [54]. However, even with modification, fundus cameras capture more reflected and scattered light compared to confocal systems [53, 55]. Scattered light from structures outside the retinal plane may falsely increase the FAF signal, a phenomena termed pseudo-autofluorescence [54].

Because the excitation spectra of fundus cameras differs from that of fluorescein angiography, the two imaging modalities can be used in any order without interference [55]. Additional advantages of fundus cameras include color imaging capability and greater resistance to motion artifact in cases of poor fixation. The single flash exposure of flood light illumination is more comfortable for the patient but produces images with low contrast, although photographers may manually acquire several images for image averaging or utilize image manipulation methods to improve contrast. Fundus cameras are less expensive than confocal scanning laser ophthalmoscopes (cSLOs), but require post-purchase modifications such as the installation of filters.

### Confocal scanning laser ophthalmoscope (cSLO)

cSLO systems utilize a system of mirrors to focus a low power laser in a two-dimensional raster pattern onto the fundus (Fig. 3b). Platforms use a blue excitation wavelength of 488 nm and detect emission wavelengths of 500–700 nm [56]. Confocal optics reduce light detection to a single optical plane, eliminating scattered light and interference from structures outside the retina, such as the crystalline lens [57]. cSLO systems offer real-time averaging, where typically nine to sixteen images are averaged to produce high-contrast and high-resolution images. There is no limit to the number of images averaged [50]. However, real-time averaging may lead to loss of information, especially in patients with poor fixation and excessive eye movement [50]. cSLO imaging cannot be preceded by fluorescein angiography, which has a similar excitation and emission spectra. A substantial fraction of the excitation beam is also absorbed by macular pigments, which have absorption spectra similar to the excitation beam wavelength [58].

Commercially available cSLO systems include the Heidelberg Spectralis^®^ and the Nidek F-10^®^. The Heidelberg retinal angiograph^®^ (HRA), now available as the Heidelberg Spectralis^®^, uses a barrier filter of 500 nm and offers 20–55 degree images. Other cSLO systems include the Zeiss prototype SM 30 4024^®^ (ZcSLO) with a barrier filter of 521 nm and the Rodenstock cSLO^®^ (RcSLO) with a barrier filter of 515 nm, though these are no longer commercially available. A comparison of the HRA^®^, ZcSLO^®^, and RcSLO^®^ demonstrates that HRA^®^ takes the brightest images, while the RcSLO^®^ captures the darkest images with the lowest contrast [59]. The level of background noise was lowest for HRA^®^ and highest for ZcSLO [59]. The Nidek F-10^®^ digital ophthalmoscope offers blue light FAF at 490 nm, fluorescein angiography, and indocyanine green angiography [60]. In addition, infrared imaging at 700 nm using Retro-mode on the Nidek F-10^®^, which relies on laterally scattered light, is more sensitive for drusen than conventional color fundus photography [61, 62].

FAF images are monochromatic and lack the color information of fundus photography. cSLO systems such as the Heidelberg Spectralis^®^ do offer reflectance-based MultiColor^®^ imaging, which can be used in conjunction with SD-OCT. This technology simultaneously acquires reflectance images at three different wavelengths: blue (486 nm), green (518 nm), and infrared (815 nm), and then superimposes the images to form a final multicolored image [63]. Recently, LaRocca and colleagues introduced a “true color” SLO using a supercontinuum laser [64]. However, these techniques use fundus reflectance imaging, and there are currently no color options for FAF.

cSLO systems can be used reliably to quantify macular pigment density, which consists of lutein, zeaxanthin, and meso-zeaxanthin [65]. With an absorption spectrum of 400–540 nm and peaking at 460 nm, macular pigments filter blue light and also act as antioxidants to protect the retina [66, 67]. Changes in macular pigment density may reflect visual function and retinal diseases such as age-related macular degeneration [68, 69]. To measure macular pigment, cSLOs such as the Heidelberg Spectralis^®^ utilize dual-wavelength autofluorescence, which uses lipofuscin autofluorescence as an indirect measure of macular pigment density. Averaged images are obtained at two excitation wavelengths, 488 and 514 nm, using a barrier filter above the macular pigment absorption wavelength (e.g. 530 nm). These images are then digitally subtracted from each other to construct a map of macular pigment density [70]. Variations of this FAF technique include using only one high absorption wavelength to obtain images, using a fundus camera instead of a cSLO, and inferring macular pigment density by comparing foveal and para-foveal autofluorescence [70]. Besides FAF, other methods used to measure macular pigment density include motion photometry, heterochromatic flicker photometry, resonance Raman spectroscopy, and fundus reflectance [65].

Although cSLO and fundus cameras use different excitation and emission wavelengths, the two systems are largely concordant on retinal pathology given the wide autofluorescent spectra of lipofuscin. While the fundus camera has lower cost and shorter image acquisition time, studies suggest superior images are obtained with cSLO in 70 % of cases [50].

### Optos ultra-widefield system

The Optomap Ultra-Widefield^®^ system by Optos^®^ combines confocal scanning laser technology with an ellipsoid mirror to achieve up to 200 degrees of view (82.5 % of retinal surface area) of the ocular fundus (Fig. 3c) [71]. The Optos^®^ system simultaneously uses two excitation wavelengths of red (633 nm) and green (532 nm) light with an emission filter of >540 nm [48]. Similar to the fundus camera, the longer wavelength spectra of this system reduces absorption by macular pigment and allows for a clear image after fluorescein angiography.

Ultra-widefield imaging allows for improved detection and analysis of many pathologic retinal conditions with peripheral findings, including diabetic retinopathy and other retinal vascular diseases, age-related macular degeneration, and myopic degeneration [72]. The Optos^®^ system has several important features, including the ability to acquire images through a native non-dilated pupil, a brief image acquisition time (250 ms), and the option of pseudocolor fundus photography [48]. However, use of the ellipsoid mirror in the Optos^®^ system distorts the peripheral retina, creating a topographic mismatch, and the view is limited superiorly and inferiorly [73]. The Optos^®^ system is also limited by lid and eyelash artifact, lack of real-time averaging, and poor contrast.

### Other modern ultra-widefield imaging systems

The Heidelberg Retinal Angiograph^®^ system can be modified for widefield imaging with the Staurenghi^®^ lens, which provides 150 degrees of view. This system is limited by lens artifact and requires placement of a contact lens by an experienced photographer [74]. More recently, Heidelberg^®^ debuted a non-contact, ultra-widefield system that provides a 105 degree view and is manually attached to the camera [75]. In contrast to the Optos^®^ ellipsoid mirror, the Heidelberg ultra-widefield^®^ system allows for high contrast, non-distorted images without lid and eyelash artifact [75]. The Heidelberg^®^ system has a smaller field of view compared to the Optos^®^ system, especially in the nasal and temporal quadrants [72].

## Fundus autofluorescence imaging in clinical practice

**Fig. 4:**
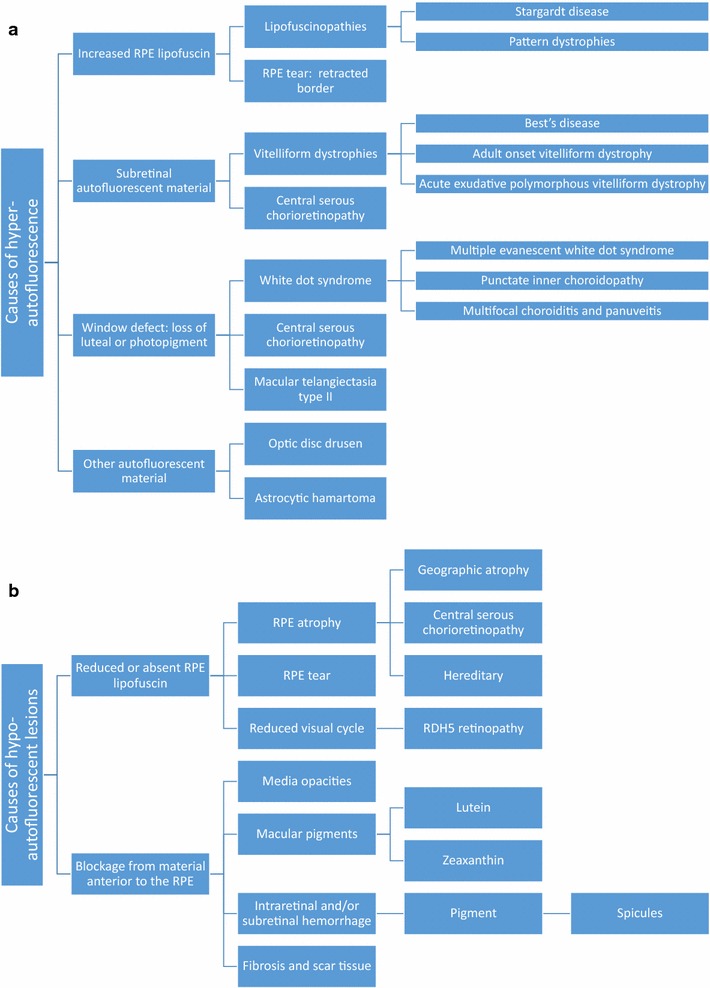
**a** Causes of hyper-autofluorescence. **b** Causes of hypo-autofluorescence

The signal intensity on FAF images depends on the concentration of lipofuscin and to a smaller degree, other fluorophores as discussed above. Lesions can be categorized as hyper-autofluorescent (Fig. 4a), hypo-autofluorescent (Fig. 4b), or iso-autofluorescent, and result from various retinal pathological mechanisms.

Autofluorescent patterns result from the complex interaction of fluorophores such a lipofuscin, which release an autofluorescent signal, and elements such as melanin and rhodopsin, which absorb the excitation beam and attenuate autofluorescence. Other structures such as retinal vessels and the crystalline lens may also influence autofluorescence through blocking and interference. Spatial variations in normal background autofluorescence reflect the distribution of lipofuscin and other fluorophores, which in turn reflect the distribution of photoreceptors and RPE cells. Meanwhile, the overall intensity of the autofluorescent signal is determined by individual factors such as age [5], light exposure [76], genotype, etc.

A large portion of hyper-autofluorescent lesions results from alterations in lipofuscin metabolism. Accumulation of lipofuscin in RPE cells, accumulation of bisretinoids in photoreceptor outer segments, and photooxidation of bisretinoids in lipofuscin can all contribute to enhanced autofluorescence [77]. Specific mechanisms vary by disease, ranging from the inability to clear bisretinoids from photoreceptor cells in Stargardt disease [78] to superimposed RPE cells in geographic atrophy (GA) of age-related macular degeneration (AMD) [79]. Accumulation of subretinal vitelliform material in Best disease, adult onset vitelliform maculopathy, and other acquired vitelliform disorders is another classic etiology of hyper-autofluorescence. Hyper-autofluorescence can also result from a window defect due to loss of rhodopsin, which normally absorbs the excitation wavelength and decreases autofluorescence. Rhodopsin attenuation and hyper-autofluorescence can occur physiologically as seen in the bleaching effect. In disease, photoreceptor degeneration and loss of rhodopsin unmasks the autofluorescent signal of the underlying RPE, creating hyper-autofluorescent lesions such as in white dot syndromes and other pathologies [80]. Fluorophores other than lipofuscin, including optic disc drusen, can also form hyper-autofluorescent lesions.

In contrast, hypo-autofluorescence can arise from decreased lipofuscin or blockage by material anterior to the RPE/photoreceptors. Perhaps the most striking example of hypo-autofluorescence due to decreased lipofuscin is the absence of autofluorescence at the site of RPE tears, where the RPE is completely denuded [81]. Other pathologies involving decreased lipofuscin include RPE atrophy in GA and visual cycle defects in fundus albipunctatus. Meanwhile, blockage can occur from any material anterior to the RPE/photoreceptors, including physiologic structures such as blood vessels and macular pigments, and pathologic structures such as media opacities, intraretinal or subretinal hemorrhage, and fibrosis.

## Age-related macular degeneration

Affecting 6.5 % of Americans over 40 years old, AMD can be associated with vision loss in both non-neovascular and neovascular forms of disease [82]. FAF can be an important tool to monitor non-neovascular AMD—especially GA, the second most important etiology of severe vision loss in AMD after choroidal neovascularization [83].

In early non-neovascular AMD, FAF may show hyper and hypo-autofluorescent areas that reveal more widespread disease than appreciated with fundoscopy or color photography. Hyperpigmented lesions may represent melanin granules, which correlate with hypo-autofluorescence, or melanolipofuscin granules, which correlate with hyper-autofluorescence [84]. Depigmented, hypo-autofluorescent areas correspond to RPE atrophy, which may signal early geographic atrophy [84, 85]. In 2005, the International FAF Classification Group defined eight phenotypic FAF patterns associated with early non-neovascular AMD: normal, minimal change, focal increase, patchy, linear, lace-like, reticular, and speckled [86].

### Drusen

Drusen have an extremely variable appearance on FAF depending on size, composition, and health of the overlying RPE and ellipsoid layer [87]. Large drusen are more likely to result in FAF changes, while small drusen may be iso-autofluorescent and remain undetected [88]. Intermediate drusen (63–125 µm in diameter) demonstrate a pattern of central hypo-autofluorescence with an annulus of hyper-autofluorescence, likely due to central RPE atrophy surrounded by abnormal RPE [89]. Cuticular drusen, associated with vitelliform macular detachment and described as multiple dense nodules creating a “stars in the sky appearance” with fluorescein angiography, appear hypo-autofluorescent with FAF [90]. Crystalline drusen also appear hypo-autofluorescent due to associated RPE atrophy [85].

Hyper-autofluorescent lesions include large, soft confluent drusen and drusenoid pigment epithelial detachments (PED) [88]. Drusenoid PEDs typically appear as a foci of hyper-autofluorescence bordered by a hypo-autofluorescent halo, but may produce intermediate to decreased signal if there is overlying RPE atrophy or fibrovascular scarring. They are associated with a high risk for disease progression [91].

First described as dot-like spots seen on blue light photography, reticular pseudodrusen are subtle accumulations of material above the RPE and visualized best on FAF, near-infrared FAF, or SD-OCT [92, 93]. They appear as small, round, elongated foci of hypo-autofluorescence bounded by interspersed hyper-autofluorescence in a reticular pattern [94]. Reticular pseudodrusen are associated with a high risk for progression to advanced disease involving choroidal neovascularization and/or geographic atrophy [95].

### Geographic atrophy

Geographic atrophy is the hallmark of advanced non-neovascular AMD. Lesions typically occur parafoveally, with foveal sparing. RPE atrophy and consequent loss of intrinsic fluorophores produces an area with a low to extinguished FAF signal with sharply demarcated borders. Lesions that include the fovea are more difficult to visualize on FAF due to low baseline concentration of lipofuscin and absorption interference by macular pigment.

**Fig. 5:**
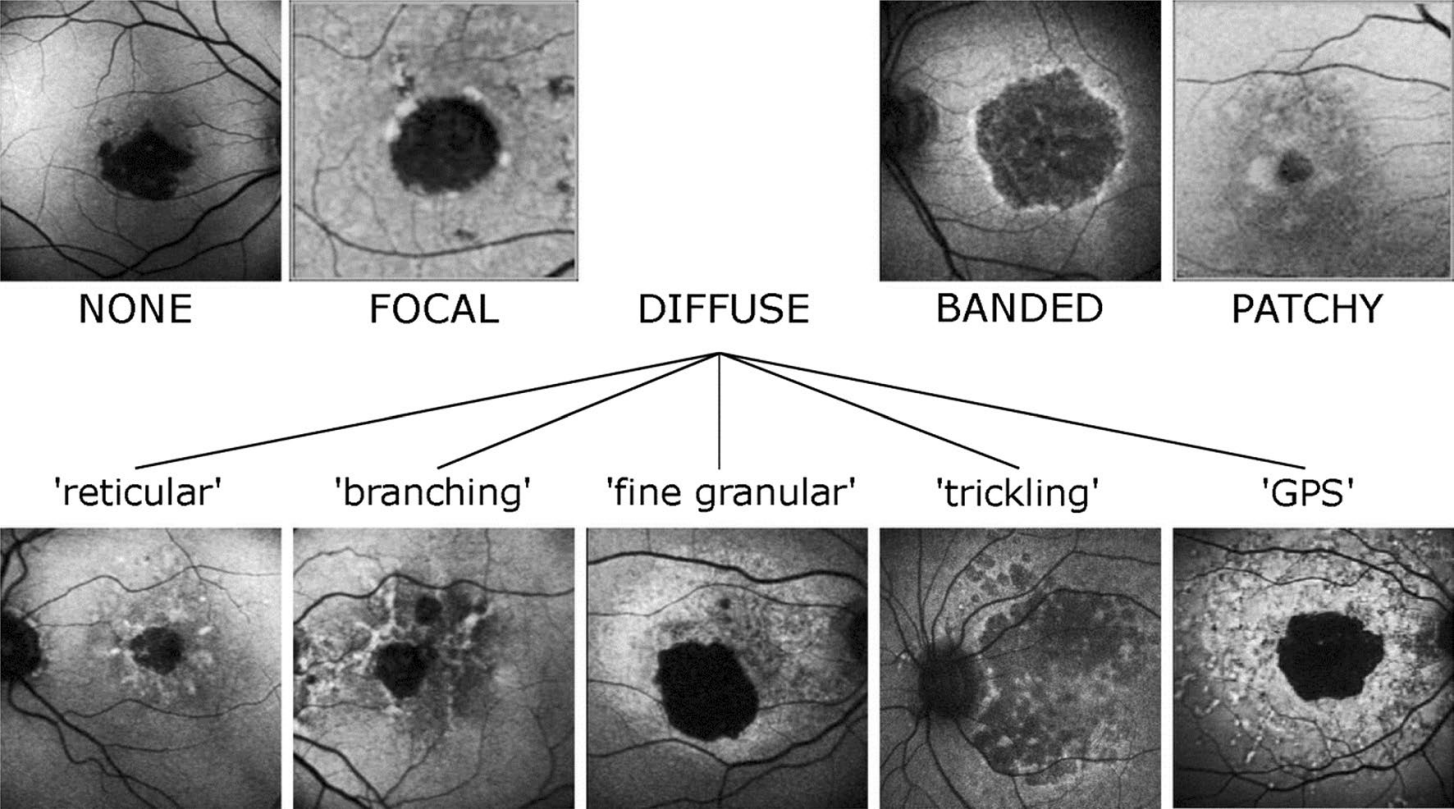
Perilesional hyper-autofluorescence patterns in geographic atrophy in age-related macular degeneration. Geographic atrophy can be classified based on perilesional phenotypic FAF patterns. The subtype may impact prognosis, with diffuse and banded phenotypes portending greater risk for progression to advanced disease. Republished with permission of Association for Research in Vision and Ophthalmology, from “A subgroup of age-related macular degeneration is associated with mono-allelic sequence variants in the ABCA4 gene”, Lars G. Fritsche; Monika Fleckenstein; Britta S. Fiebig; Steffen SchmitzValckenberg; Almut BindewaldWittich; Claudia N. Keilhauer; Agnes B. Renner; Friederike Mackensen; Andreas Maner; Daniel Pauleikhoff; Christine Adrion; Ulrich Mansmann; Hendrik P. N. Scholl; Frank G. Holz; Bernhard H. F. Weber, vol 53, 2012; permission conveyed through Copyright Clearance Center, Inc

Geographic atrophy may be surrounded by perilesional hyper-autofluorescence, which represent areas of ongoing RPE cell dysfunction, vertically superimposed RPE cells, and variable progression to atrophy [79, 96]. Phenotypes of perilesional hyper-autofluorescence include: none, focal, diffuse, banded, and patchy (Fig. 5). The diffuse phenotype is further delineated into reticular, branching, trickling, fine granular, and fine granular with peripheral punctate spots (GPS) patterns. Diffuse (especially the trickling pattern) and banded phenotypes are associated with a higher risk for disease progression. Given the potential for understanding and predicting disease progression, FAF evaluation of atrophic AMD is an important clinical and research modality [97].

### Choroidal neovascularization

Neovascular AMD is defined by the presence of choroidal neovascularization, which is located either above the RPE (type 2), or under the RPE (type 1). Type 3 neovascularization is located intraretinally and originates from the deep retinal capillary plexus [98].

Early choroidal neovascularization is not readily detectable on FAF, reflecting intact RPE and photoreceptor layers [99]. Classic choroidal neovascularization appears hypo-autofluorescent due to blockage of the RPE by the type 2 fibrovascular complex in the subretinal space [100]. Occult type 1 neovascularization is also hypo-autofluorescent due to associated atrophy of the overlying RPE. Choroidal neovascularization may be bordered by hyper-autofluorescence in 38 % of cases due to associated RPE proliferation or photoreceptor loss resulting in a window defect [101]. Hemorrhages and exudates are initially hypo-autofluorescent due to excitation light absorption, but then may become hyper-autofluorescent after undergoing organization.

Fundus autofluorescence patterns in non-neovascular AMD may predict the development of choroidal neovascularization. Batoglu et al. found that the patchy pattern of early non-neovascular AMD had the strongest correlation with progression to neovascular AMD, with 30.4 % of eyes developing choroidal neovascularization the mean follow-up period of 29.2 months [102, 103]. Linear and reticular patterns were also associated with increased risk for choroidal neovascularization [102].

### RPE tears

RPE tears are a well-known complication of neovascular AMD, most commonly associated with large (greater than 600 microns in height) fibrovascular PEDs [104]. RPE tears can occur spontaneously or following photodynamic therapy or anti-VEGF therapy due to accelerated contraction of the neovascular complex exerting traction on the RPE monolayer [105, 106].

**Fig. 6:**
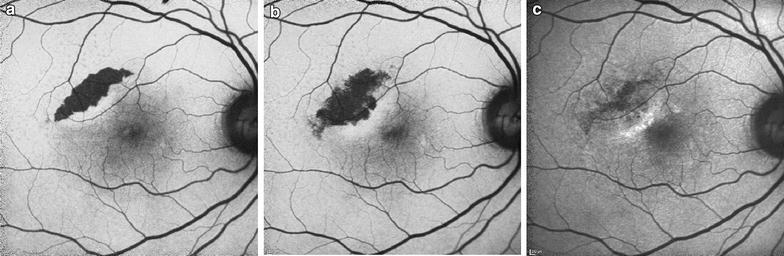
Sequential fundus autofluorescence of an RPE tear. RPE tears (**a**) appear as well-demarcated central hypo-autofluorescence due to absent RPE with adjacent irregular hyper-autofluorescence corresponding to the retracted edges of RPE. Serial images obtained 3 weeks (**b**) and 1 year (**c**) later show resurfacing and remodeling of the lesion, with centripetal recovery of autofluorescence extending from the borders

**Fig. 7:**
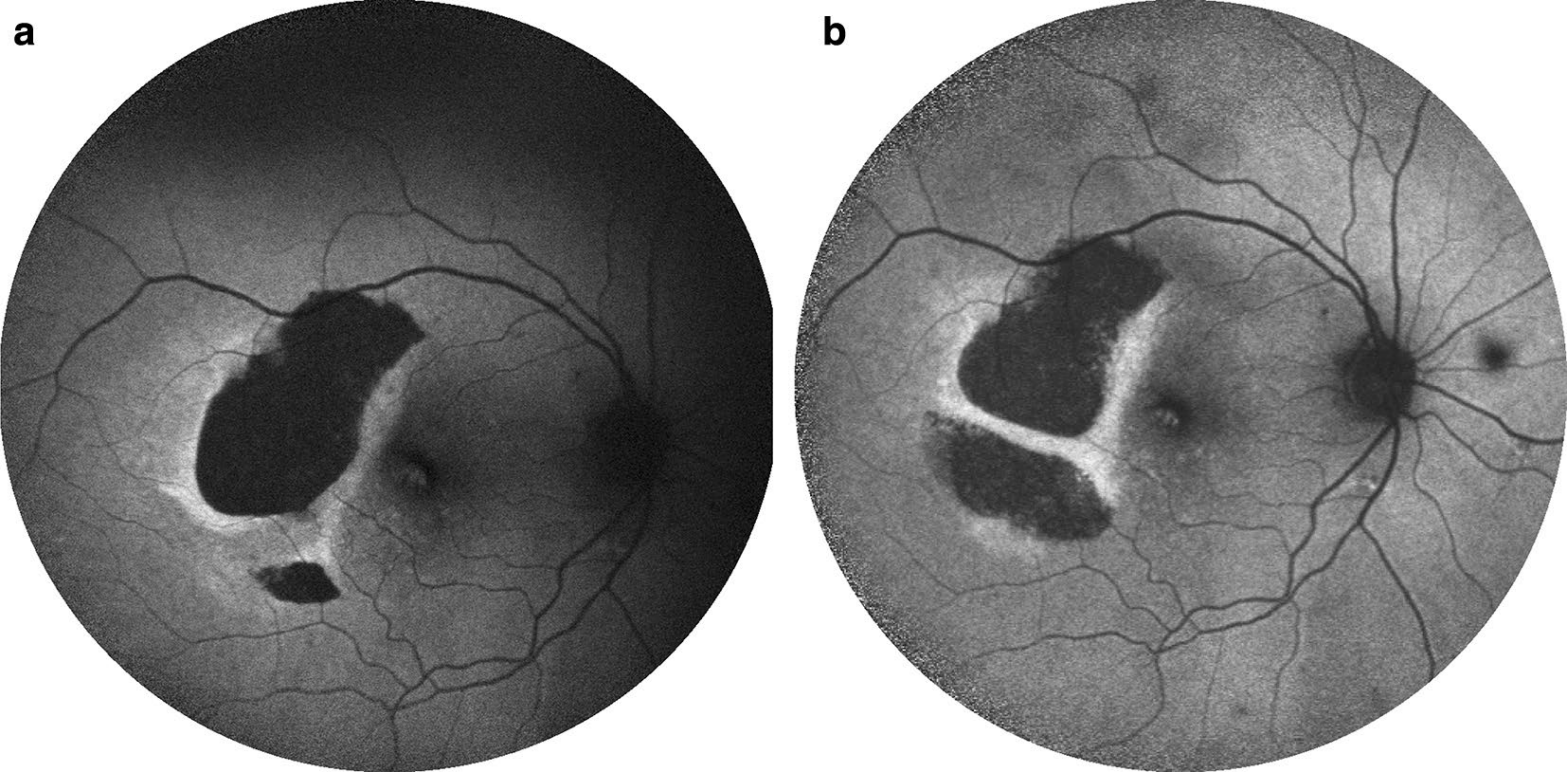
Sequential fundus autofluorescence of an RPE tear treated with aflibercept. RPE tears (**a**) appear as a well-demarcated central hypo-autofluorescence due to absent RPE with adjacent irregular hyper-autofluorescence corresponding to the retracted edges of RPE. The patient received anti-VEGF therapy with half-dose aflibercept. Over time (**b**), the lesion shows evidence of slight remodeling and resurfacing, with early centripetal recovery of autofluorescence. Note the inferior extension of the tear. Photo courtesy of Nagiel A, Sadda S, Schwartz S, Sarraf D. “Resolution of a giant pigment epithelial detachment with half-dose aflibercept.” Retinal Cases and Brief Reports. Vol 9, Issue 4, 269–272. Promotional and commercial use of the material in print, digital or mobile device format is prohibited without the permission from the publisher Wolters Kluwer Health. Please contact healthpermissions@wolterskluwer.com for further information

RPE tears appear as a well-demarcated area of hypo-autofluorescence due to absent RPE, with adjacent hyper-autofluorescence in the form of rolled redundant RPE. Over time, tears remodel and resurfacing occurs, with recovery of autofluorescence extending centripetally from the borders toward the center (Fig. 6). The process of resurfacing correlates with visual improvement and may benefit from treatment with anti-VEGF, though studies differ (Fig. 7) [105, 107, 108].

### Differential diagnosis of age-related macular degeneration

The wide variation in clinical presentation and severity of AMD, as well as its considerable overlap with other macular dystrophies, presents an interesting diagnostic challenge. Although drusen are the hallmark of AMD, they are not pathognomonic for the disease. Small drusen <63 µm represent normal aging rather than AMD [109]. Drusen should be differentiated from the irregular flecks of late onset Stargardt disease, which are characterized by intense hyper-autofluorescence on FAF. Drusen should also be differentiated from vitelliform lesions such as in Best dystrophy and adult onset vitelliform macular dystrophy, discussed below. Macular dystrophies that present with drusen or drusen-like deposits include Malattia Leventinese, Sorsby fundus dystrophy, and North Carolina macular dystrophy, all of which have an earlier age of onset and autosomal dominant inheritance. Geographic atrophy can resemble central areolar choroidal dystrophy, late onset cone dystrophy, and central serous chorioretinopathy [110] and can be differentiated with the aid of FAF.

## Central serous chorioretinopathy

**Fig. 8:**
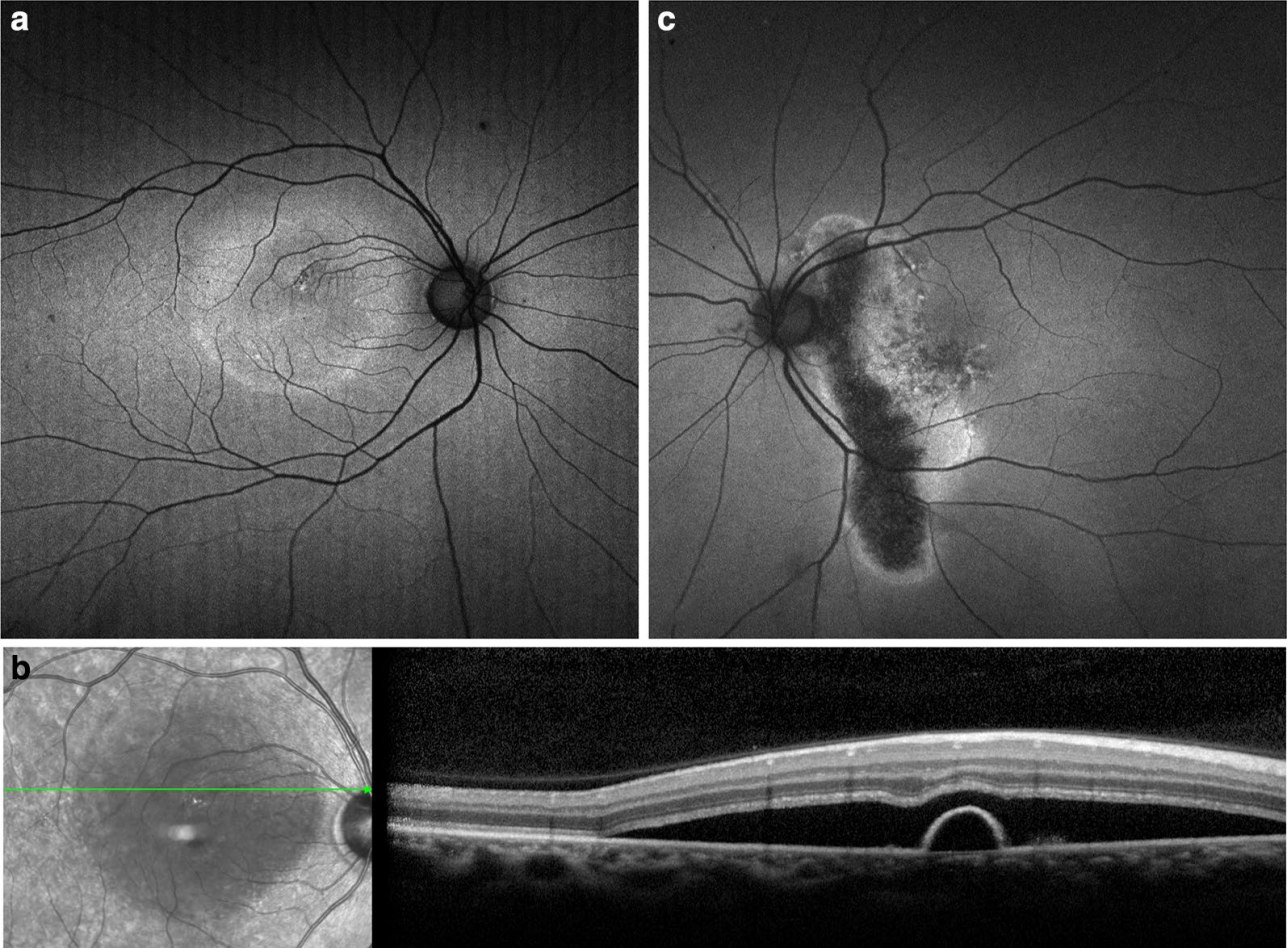
Fundus autofluorescence of central serous chorioretinopathy. FAF (**a**) of a son with acute exacerbation of CSCR shows macular detachment due to acute CSCR, with hyper-autofluorescent material at the margin and inferior region of the detachment. SD-OCT (**b**) of the lesion shows a serous retinal detachment associated with a small pigment epithelial detachment. FAF of the patient’s asymptomatic father (**c**) incidentally revealed an atrophic hypo-autofluorescent gravitational tract from chronic inactive CSCR with hyper-autofluorescent margins

CSCR most often presents as a serous retinal detachment that results from choroidal dysfunction and idiopathic leakage from the RPE into the subretinal space. During the initial presentation of CSCR, 72–96 % of cases show hypo-autofluorescence corresponding to the focal leakage site on fluorescein angiogram and to the area of serous retinal detachment, due to blockage by subretinal fluid [111–113]. A subset of patients may show localized RPE detachment, corresponding with focal hyper-autofluorescence. As the disease progresses, subsequent FAF images show granular hyper-autofluorescence, with increased number and size of hyper-autofluorescent dots corresponding to subretinal precipitates on OCT (Fig. 8a, b) [113]. This material represents photoreceptor debris and macrophages accumulating in the subretinal space due to separation of the neurosensory layer from the RPE [114, 115]. The hyper-autofluorescent precipitates gravitate inferiorly or collect at the borders of the detachment. In chronic cases lasting longer than 6 months, 85 % showed hypo-autofluorescent atrophic gravitational tracts that correlate to prior dependent subretinal fluid (Fig. 8c) [111, 114].

Near-infrared FAF is more sensitive for CSCR than conventional short-wavelength FAF, with short-wavelength FAF revealing 83.2 % of abnormalities compared to 100 % by near-infrared FAF [116]. Ultra-widefield FAF can aid in the diagnosis and monitoring of CSCR [117]. Pang et al. found that 57 % of CSCR cases had extensive involvement of the peripheral retina. Abnormal hyper-autofluorescent lesions in the periphery could indicate active disease and warrants further evaluation by SD-OCT and indocyanine green angiography [118].

## Macular dystrophies

### Stargardt disease

Stargardt macular degeneration is the most common hereditary juvenile macular dystrophy. The disorder most commonly results from an autosomal recessive mutation in the ABCA4 gene, leading to defective outer segment degradation, lipofuscin accumulation, and central degeneration of the RPE and photoreceptor layer. Rare cases of autosomal dominant Stargardt disease result from mutations in the Elongation of Very Long Chain Fatty Acids (ELOVL4) gene [119]. Clinically, Stargardt disease presents as foveal atrophy surrounded by flecks of yellow deposits, peripapillary sparing, and associated central vision loss [120].

**Fig. 9:**
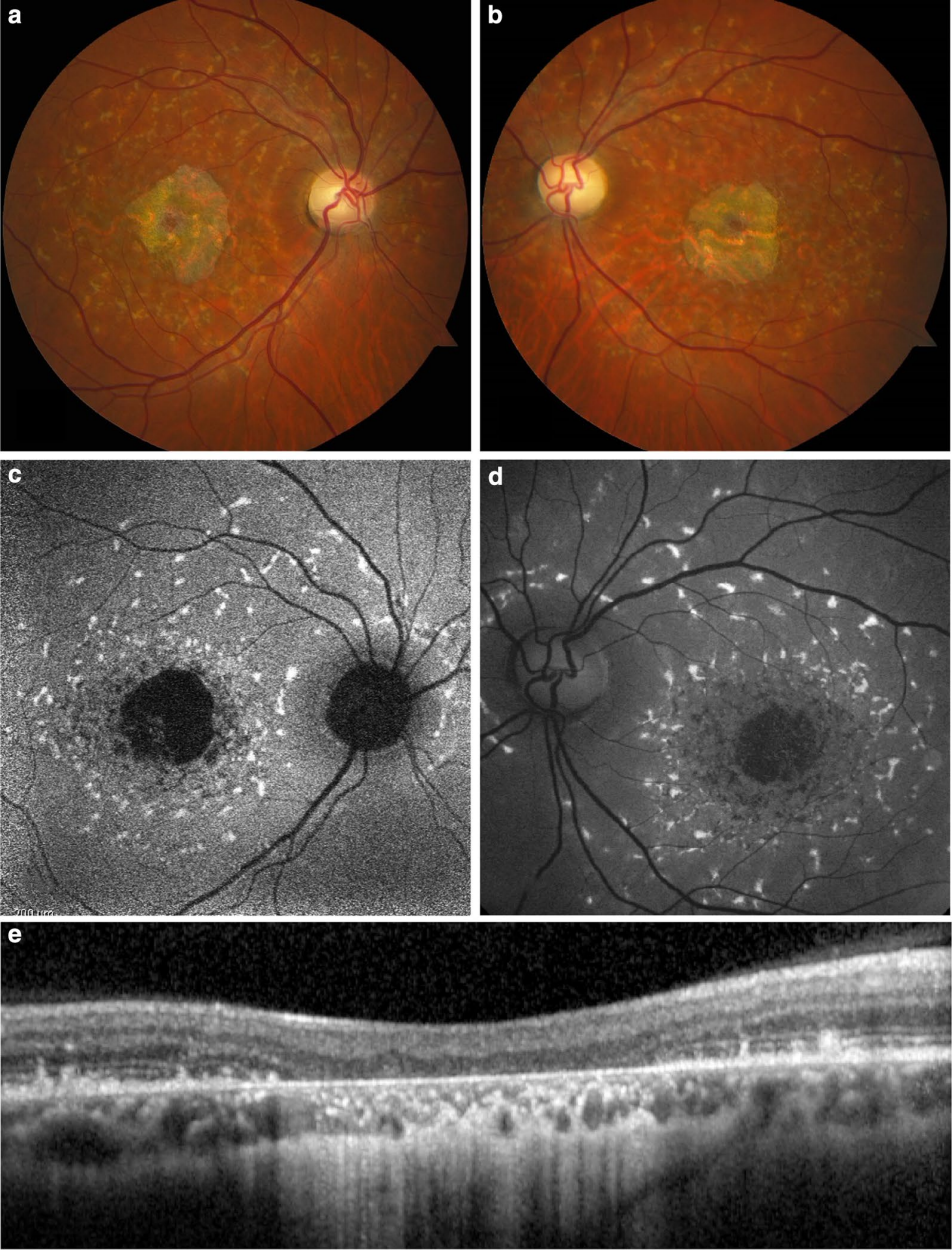
Fundus autofluorescence of Stargardt disease. While fundus photography (**a**, **b**) and clinical examination may have non-specific findings, fundus autofluorescence (**c**, **d**) shows hyper-autofluorescent flecks with peripapillary sparing characteristic of the disease. The flecks surround a hypo-autofluorescent central macula corresponding to significant chorioretinal atrophy on SD-OCT (**e**)

Cideciyan et al. synthesized a six stage model of disease pathogenesis that correlates well with FAF findings [121]. Early stages may demonstrate a general increase in lipofuscin and thus increased autofluorescent signal [120, 122]. Intermediate stages show a high variation in mean intensity and texture (microscopic spatial variation in intensity) [121]. The disease then progresses to a pattern of chorioretinal atrophy resulting in macular hypo-autofluorescence, surrounded by hyper-autofluorescent flecks (Fig. 9) [123]. Peripapillary sparing of the hyper-autofluorescent flecks is highly suggestive of Stargardt disease. In advanced stages, complete degeneration and diffuse atrophy of RPE cells and photoreceptor death result in hypo-autofluorescence and vision loss [123].

FAF may show areas of early atrophy and flecks not otherwise appreciated with fundus photography, suggesting its utility in detecting early disease [123]. In addition, FAF correlates well with visual function, with normal macular autofluorescence associated with normal electroretinography findings and good vision [120].

### Best macular dystrophy

An early-onset macular dystrophy resulting in central vision loss, Best disease arises from autosomal dominant mutations in the BEST1 gene, encoding bestrophin-1. Shed photoreceptor debris and lipofuscin accumulate in the subretinal space, resulting in bilateral yolk-like lesions in the macula [124–126].

The progression of disease is classically separated into five stages of progression: previtelliform, vitelliform, pseudohypopyon, vitelliruptive, and atrophic, with progression at variable rates. In a retrospective, observational study, Querques et al. found that previtelliform lesions showed zero to minimal hyper-autofluorescence. The vitelliform stage had well-circumscribed, homogenous hyper-autofluorescence in the macula. The pseudohypopyon stage showed a gravitational layer of hyper-autofluorescence settling under iso-autofluorescent fluid. The vitelliruptive stage showed a dark lesion bordered by condensations of hyper-autofluorescent material. The atrophic stage was characterized by diffuse, decreased signal due to chorioretinal atrophy [125].

Parodi et al. characterized six different phenotypes of Best disease with FAF, including normal, hyper-autofluorescent, hypo-autofluorescent, patchy, multi-focal, and spoke-like patterns. The patchy subtype occurred most frequently, followed by the hyper-autofluorescent, then hypo-autofluorescent patterns. These findings did not correlate with the stages of progression or best corrected visual acuity [124].

### Acute exudative polymorphous vitelliform maculopathy

Occurring as an idiopathic [127] or paraneoplastic syndrome [128, 129], acute exudative polymorphous vitelliform maculopathy (AEPVM) likely results from inflammatory or immune-mediated RPE dysfunction leading to lipofuscin accumulation and exudative retinal detachments [130]. AEPVM is characterized by multifocal, yellow-white, subretinal lesions associated with serous retinal detachments. These lesions are similar to the vitelliform deposits of Best disease, although genetic testing is negative for BEST1 mutations [131, 132]. FAF shows intense hyper-autofluorescence of the subretinal vitelliform material, which tends to gravitate inferiorly [133]. Lesions resolve gradually over weeks, with a corresponding decrease in autofluorescence [130].

### Pattern dystrophies

Pattern dystrophies refer to a collection of late-onset, symmetric macular dystrophies with a clinically stable, benign course. Subtypes include adult onset vitelliform dystrophy, “butterfly” pigment dystrophy, reticular dystrophy, multifocal pattern dystrophy simulating fundus flavimaculatus, and fundus pulverulentus [134]. Pattern dystrophies are commonly associated with autosomal dominant mutations in the PRPH2 gene (formerly known as RDS, retinal degeneration slow gene) on chromosome 6, which codes for a membrane glycoprotein on rod and cone photoreceptor outer segments [135]. As a result, yellow, orange, or gray material accumulates subretinally or at the level of the RPE, translating to hyper-autofluorescent lesions [136].

Adult-onset vitelliform dystrophy predominantly results from mutations in the PRPH2 gene and less commonly the BEST1 genes [137]. This disease presents as small bilateral, subfoveal vitelliform lesions with a central clump of pigment that may evolve to RPE atrophy [138]. These vitelliform lesions emit increased autofluorescence [139, 140], while subsequent RPE atrophy results in hypo-autofluorescence [138]. Furino et al. described three FAF patterns, including patchy, ring-like focal, and linear patterns, but did not find a correlation with progression or visual function [141]. Around the same time, a slightly larger study by Parodi et al. identified three separate FAF patterns that progressed from normal, to focal, then to patchy hyper-autofluorescence, which correlated with visual acuity and retinal sensitivity. Of note, near-infrared FAF, which detects melanin, had a higher sensitivity than conventional short-wavelength autofluorescence, visualizing abnormalities in 100 versus 86 % of cases [142].

Multifocal pattern dystrophy simulating fundus flavimaculatus presents as irregular yellow flecks that are concentrated at the posterior pole and vascular arcades and that show hyper-autofluorescence on FAF. Classic “dot and halo” lesions appear as central hypo-autofluorescence surrounded by a hyper-autofluorescent halo [99]. Despite a similar appearance, multifocal pattern dystrophy can be differentiated from Stargardt disease by its autosomal dominant inheritance pattern, adult-onset, benign course (geographic atrophy is atypical with pattern dystrophy), and lack of a dark choroid [143].

Other pattern dystrophies, especially “butterfly” lesions, reticular dystrophy, and fundus pulverulentus have not been well characterized on FAF imaging. However, the FAF findings can be remarkable and are more obvious than can be appreciated with examination by color photography. Moreover, FAF is a much less invasive procedure than fluorescein angiography and therefore is an important tool to reliably determine the diagnosis of macular dystrophies such as pattern dystrophy. Note that there is a high degree of phenotypic variability with pattern dystrophy. Multiple patterns may manifest with the same mutation, or even in the same patient [99]. Further investigations to understand epigenetic and environmental effects on phenotype will help elucidate disease pathogenesis and identify potential treatments.

## Retinitis pigmentosa

Retinitis pigmentosa (RP) refers to a genetically heterogeneous group of retinal dystrophies characterized by the degeneration of rod photoreceptors. Mutations in the RHO (rhodopsin) gene are most common in autosomal dominant RP, mutations in the USH2A (Usher’s type 2) gene are most common in autosomal recessive RP, and RPGR and RP2 gene mutations are most common in X-linked RP. Patients may exhibit an annular scotoma in the mid-periphery that progressively extends towards the fovea, eventually involving cone photoreceptors and impinging on central vision. Electroretinography (ERG) is the gold standard modality for diagnosis and monitoring of disease progression, but loses clinical value in advanced disease [144]. As such, FAF is a viable supplemental imaging modality to monitor RP and correlate phenotype with genotype [145].

**Fig. 10:**
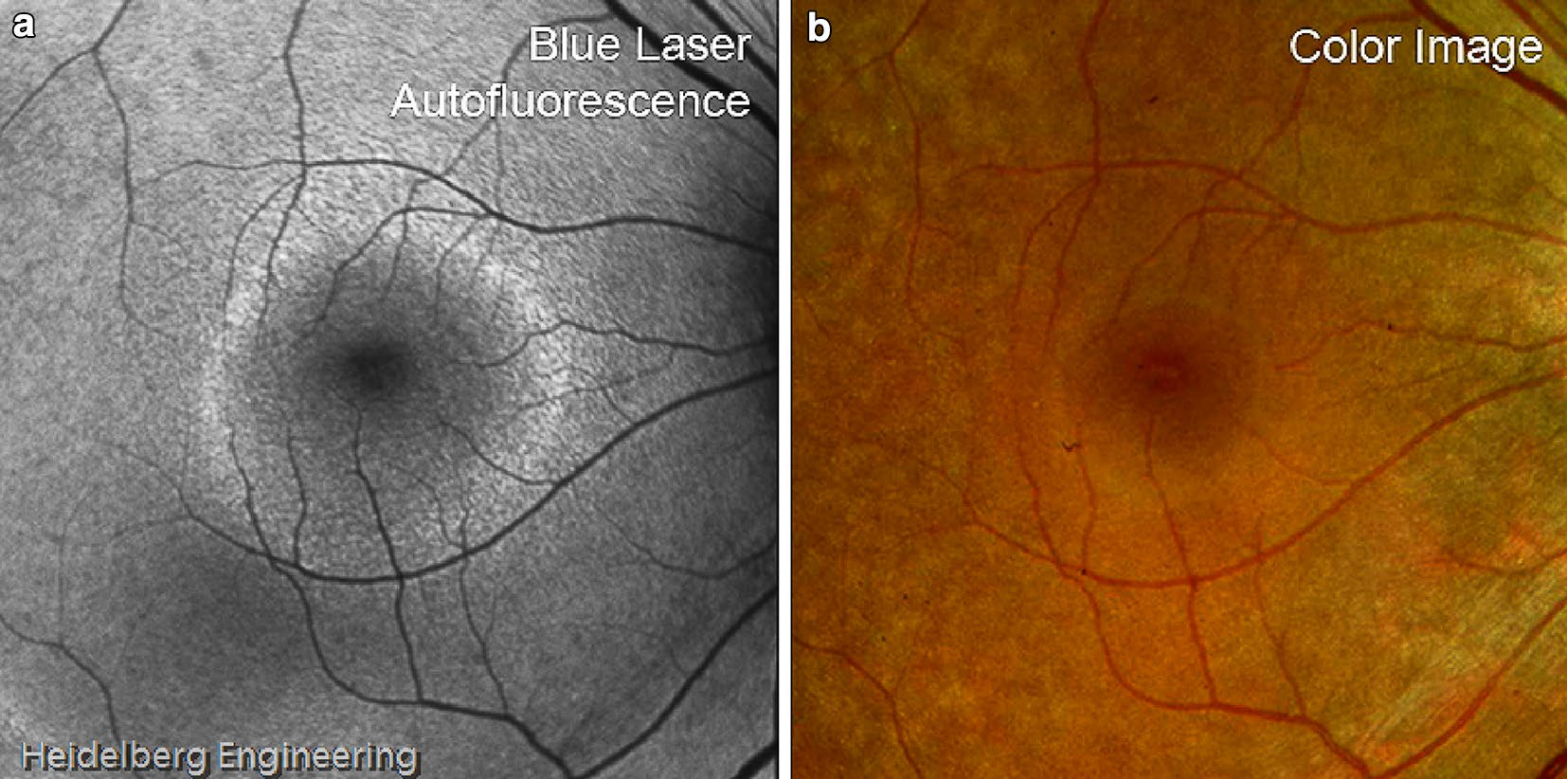
Robson-holder ring in retinitis pigmentosa. FAF (**a**) of retinitis pigmentosa shows an area of normal preserved retina at the posterior fundus bordered by a hyper-autofluorescent Robson-holder ring. Mottled hypo-autofluorescence outside the ring represents photoreceptor degeneration. These details are visible on FAF but not on fundus photography (**b**). Image courtesy of Heidelberg Engineering

A retrospective, observational case series by Murakami et al. identified three subsets of RP on FAF, where 59 % of patients had a hyper-autofluorescent parafoveal ring not visible on funduscopic exam, 18 % had abnormal central hyper-autofluorescence extending centrifugally from the fovea, and 24 % had neither pattern [146]. The hyper-autofluorescent ring, known as the Robson-Holder ring, corresponds to the border of inner/outer segment junction disruption (Fig. 10). OCT analysis shows complete photoreceptor loss outside of the ring, with the external limiting membrane in direct apposition to the RPE [147, 148]. The ring itself corresponds to outer segment dysgenesis and lipofuscin production, while normal retina lies within the ring [144, 149–151]. Spatial preservation of retinal sensitivity as measured by multifocal ERG correlates with the radius of the autofluorescent ring, indicating intact retinal sensitivity inside the ring but none outside [152]. In addition, the size of the ring correlates with visual function as measured by both kinetic and automated perimetry; the more the ring encroached centrally, the more constricted the visual field [153]. Serial imaging of this hyper-autofluorescent ring may help determine the stability or rate of progression of the disease, providing a useful tool for delineating prognosis in different patients [147].

Similar hyper-autofluorescent rings are also seen in several other retinal dystrophies, including Leber congenital amaurosis (LCA), bull’s eye maculopathy, X-linked retinoschisis, Best macular dystrophy, cone dystrophy, and cone-rod dystrophy [149]. This shared phenotype on FAF suggests an underlying common mechanism for the pathogenesis of retinal dystrophies.

## Choroideremia

Choroideremia is an X-linked recessive defect in the CHM protein, which encodes the rab escort protein (REP1). Beginning in adolescence, male carriers experience night blindness and visual field constrictions due to centripetal atrophy of the choroid, RPE, and photoreceptor layer, though the macula is spared [154].

**Fig. 11:**
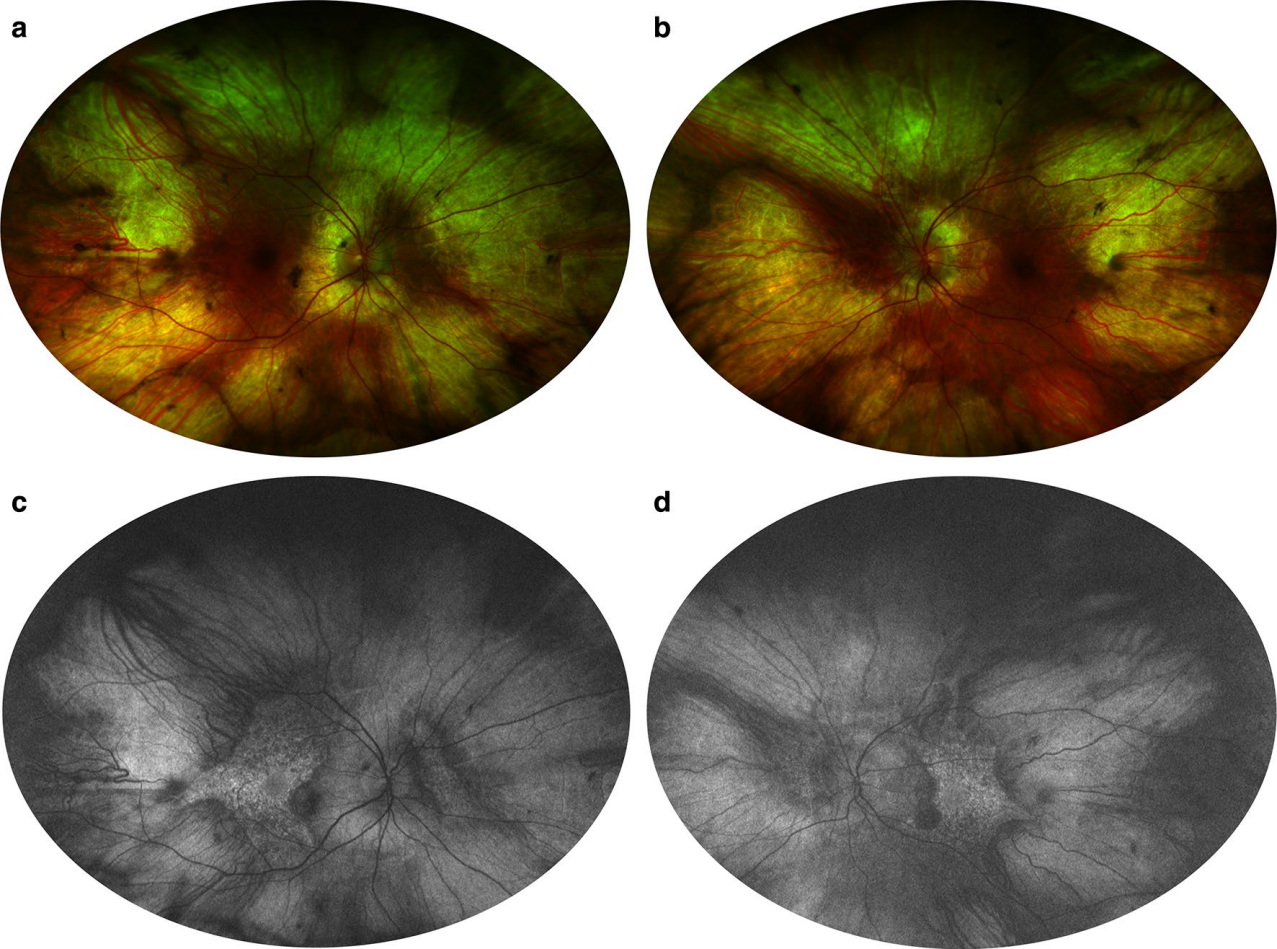
Fundus autofluorescence of choroideremia. Wide-field color fundus photography (**a**, **b**) shows a pale fundus with zones of choroidal atrophy in the mid-periphery, revealing the sclera underneath. Wide-field fundus autofluorescence (**c**, **d**) demonstrates corresponding zones of hyper-autofluorescence given the autofluorescent properties of the sclera. Note the central stellate island of preserved choriocapillaris and RPE in each eye that is characteristic of choroideremia. Photo credit: Srinivas Sadda, MD

FAF shows bilateral, symmetric, midperipheral zones of hypo-autofluorescence due to RPE atrophy, which have scalloped edges with a preserved area of central stellate autofluorescence (Fig. 11) [155]. The atrophic zones extend with age and eventually involve the fovea [156]. Recent work on gene therapy for choroideremia has shown promising preclinical results and is currently undergoing clinical trials, with the first treatment administered in 2011 [157, 158]. FAF is an accurate structural marker that correlates with visual acuity and age, and may help identify candidates for gene therapy [154].

Interestingly, FAF of asymptomatic female carriers of the CHM mutation show a peripheral speckled pattern of hyper-autofluorescence corresponding to lipofuscin accumulation from photoreceptor degeneration and hypo-autofluorescence due to RPE atrophy [156, 159, 160]. In conjunction with genetic testing, FAF is a useful for evaluating female relatives of affected patients.

## Fundus albipunctatus

Fundus albipunctatus results from an autosomal recessive defect in RDH5, which encodes a retinol dehydrogenase in the RPE [161]. Patients are unable to oxidize 11-*cis*-retinol to 11-*cis*-retinal, and this impairment of rhodopsin recycling delays the regeneration of photoreceptor pigments, manifesting as delayed dark adaptation and stationary night blindness [162]. Similar to Stargardt disease, fundus albipunctatus presents as a flecked retina syndrome, with white-yellow subretinal spots in the midperiphery on funduscopy.

**Fig. 12:**
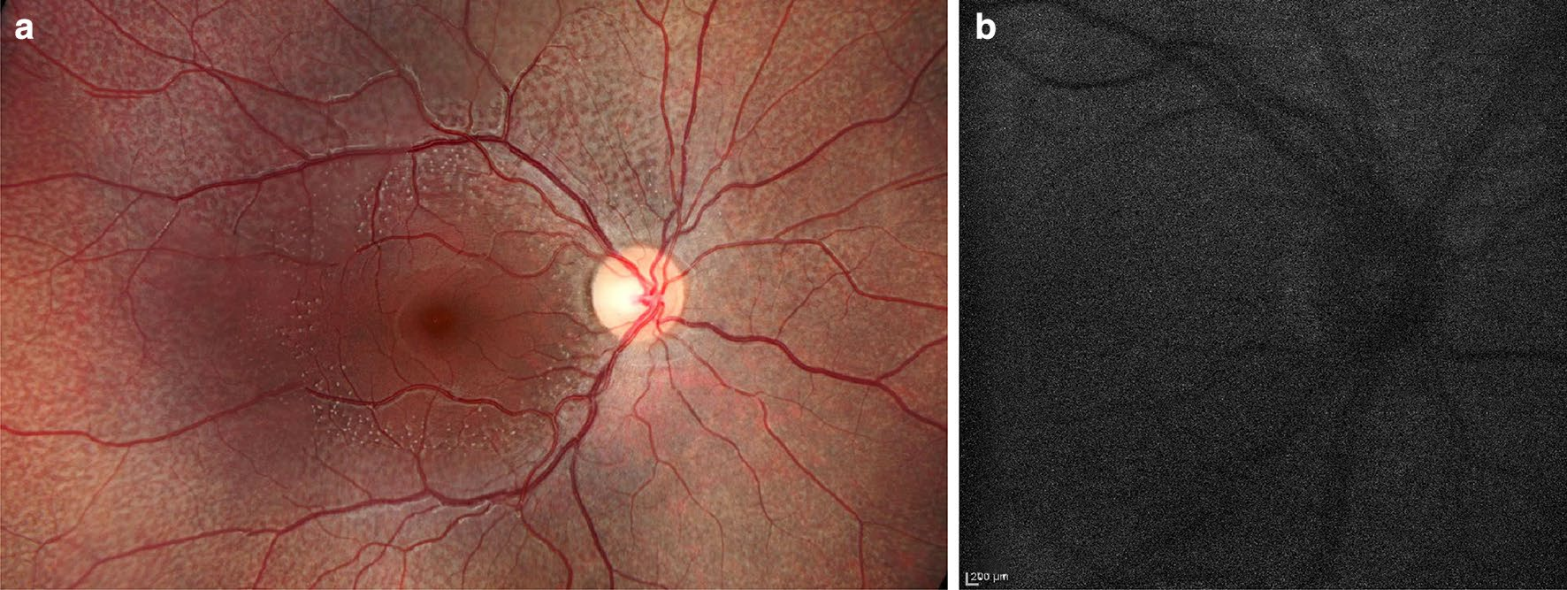
Fundus autofluorescence of fundus albipunctatus. Fundus photography (**a**) demonstrates multifocal white flecks in the midperiphery with macular sparing. FAF (**b**) shows profoundly decreased background autofluorescence and a grainy resolution. Photo credit: Sam Yang, MD

As a hereditary defect in the visual cycle, fundus albipunctatus causes decreased lipofuscin production and severely attenuated background autofluorescence [163]. Images appear grainy [164]. FAF may also show hyper-autofluorescent foci corresponding to the white dots, though not all lesions produce autofluorescence (Fig. 12) [165]. SD-OCT through these lesions reveal dome shaped hyperreflective material extending from the RPE past the photoreceptor layer [156]. Hyper-autofluorescent crescents or concentric parafoveal rings have also been reported [165].

Fundus albipunctatus must be differentiated from diseases with similar presentations. Retinitis punctata albescens is also associated with white punctate retinal lesions on the retina and night blindness. This disease is due to an autosomal recessive mutation in RLBP1 encoding CRALBP, which binds to 11-*cis*-retinol and 11-*cis*-retinal during the visual cycle [166]. However, retinitis punctata albescens is a severe progressive retinal dystrophy with narrowed vessels, pigmentary degeneration, and visual field loss [166]. Meanwhile, RPE65 encodes an isomerase which acts one step upstream of retinol dehydrogenase in the visual cycle and also results in decreased background autofluorescence on FAF [167]. In contrast to fundus albipunctatus, mutations in RPE65 result in a more severe disease course and are associated with LCA and early onset rod-cone dystrophy [168].

## White dot syndromes

White dot syndromes refer to a diverse spectrum of inflammatory chorioretinopathies characterized by multifocal, small, yellow-white lesions scattered throughout the posterior pole and/or peripheral fundus. Despite this shared phenotypic presentation, the individual diseases are not related in pathogenesis, management, or prognosis [169].

### Multiple evanescent white dot syndrome (MEWDS)

This disease affects young, healthy women 20–50 years old and manifests as a self-limited, unilateral presentation with multifocal 100–200 µm white spots scattered in the paramacular and mid-peripheral fundus [170].

**Fig. 13:**
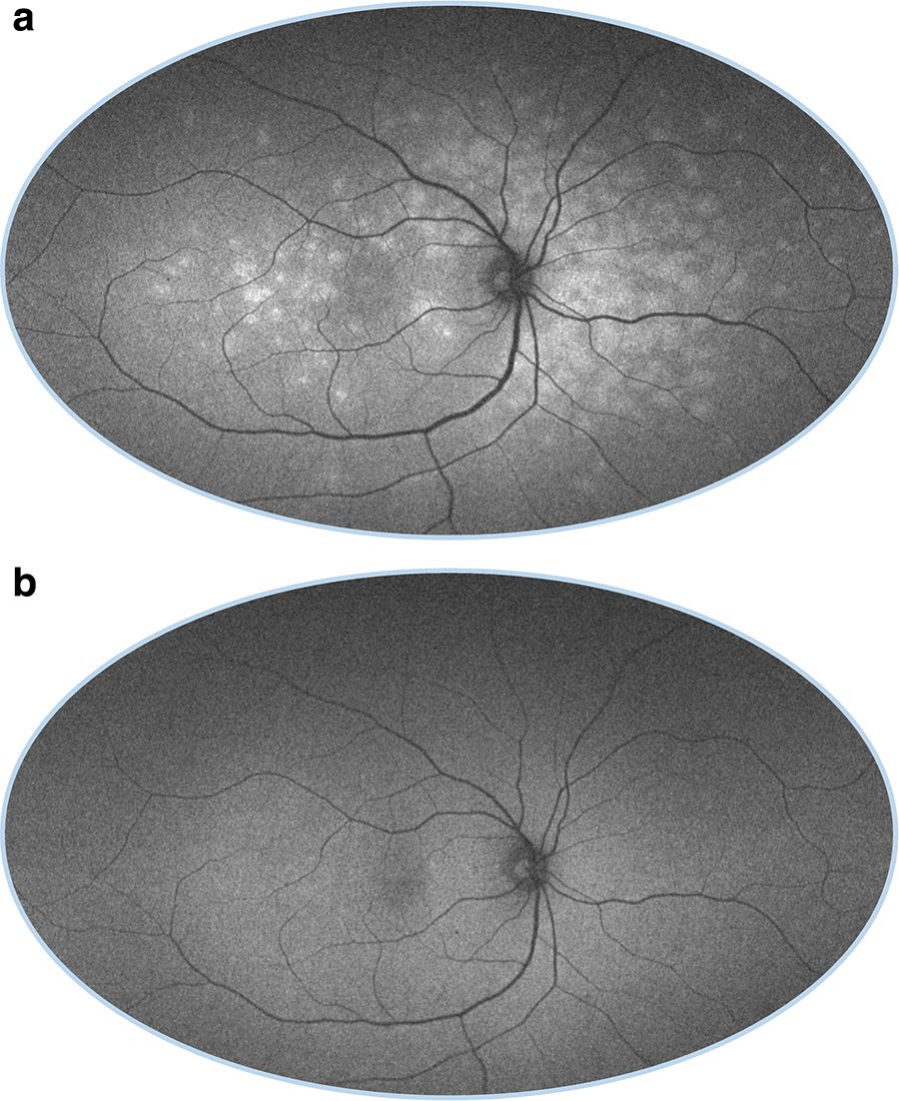
Ultra-widefield imaging of multiple evanescent white dot syndrome (MEWDS). Ultra-widefield FAF of active disease (**a**) with multifocal hyper-autofluorescence and resolution (**b**) 6 weeks later

On FAF, the multifocal lesions have increased signal strength due to photoreceptor loss and unmasking of natural RPE autofluorescence (Fig. 13) [80, 171]. Hypo-autofluorescent foci at the fovea correspond with foveal granularity on exam and accumulation of hyper-reflective material interdigitating between the RPE and photoreceptor layer on OCT [170]. FAF reveals more lesions than on clinical exam or FA, though less than seen on indocyanine green angiography [172, 173], and FAF may be used in lieu of FA and ICG due to its high sensitivity, greater ease of use, and non-invasive nature of administration.

### Punctate inner choroidopathy (PIC)

A diagnosis of exclusion, PIC refers to the presence of multifocal choroidal lesions in the absence of uveitis or presumed ocular histoplasmosis (POHS). This disease tends to occur in young, myopic women and is associated with the development of choroidal neovascularization and subsequent vision loss [174].

Active PIC lesions present as sub-RPE chorioretinal nodules, which may display minimal hyper-autofluorescence or become hypo-autofluorescent spots as the nodules break through the RPE [175]. These hypo-autofluorescent spots often have a hyper-autofluorescent margin, corresponding to a window defect of surrounding photoreceptor loss on SD-OCT [171, 175]. Meanwhile, the majority of atrophic lesions appear as isolated hypo-autofluorescent spots. A subset of patients present with hypo-autofluorescent spots associated with diffuse hyper-autofluorescent patches, 90 % of which were identified as concurrent MEWDS on multimodal imaging [171]. Similar to the choroidal neovascularization seen in ARMD, choroidal neovascularization in PIC presents as foci of slight hypo- or hyper-autofluorescence with a surrounding hyper-autofluorescent ring [175]. Additional reports have found that FAF and ultra-widefield FAF can be useful for detecting changes in eyes with PIC that are not evident on clinical exam [176, 177].

## Drug toxicity

### Hydroxychloroquine toxicity

Hydroxychloroquine is an inexpensive and well-tolerated anti-inflammatory agent. However, the risk for ocular toxicity rises sharply with cumulative dose, especially with doses over 1000 g [178]. In addition, because the drug is not retained in fatty tissues, individuals who are obese or have short stature are at risk for overdosage [179].

**Fig. 14:**
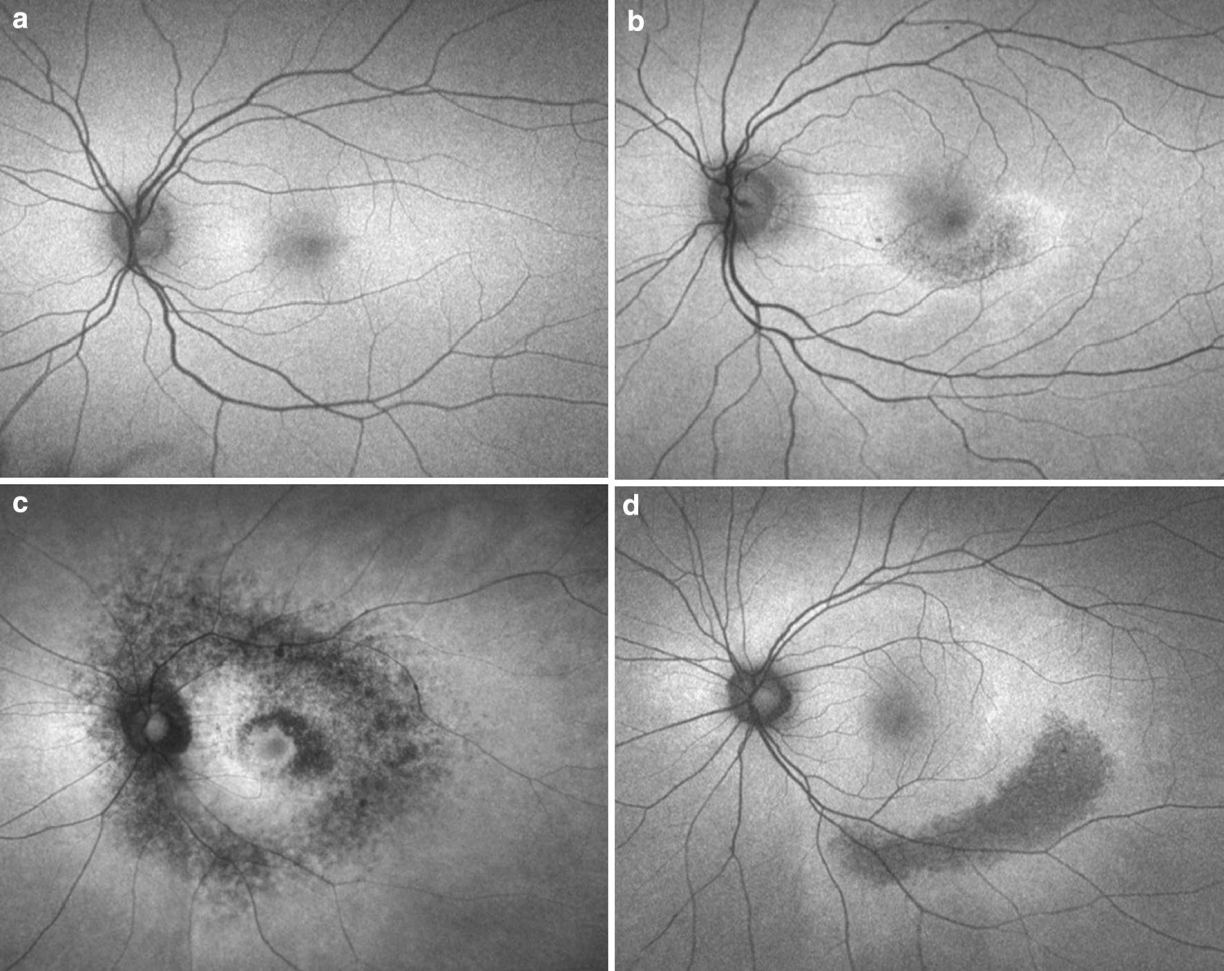
Spectrum of hydroxychloroquine toxicity on FAF. Compared to a normal fundus (**a**), hydroxychloroquine toxicity may manifest in a parafoveal pattern (**b**), mixed pattern (**c**), or pericentral pattern of characteristic FAF abnormalities (**d**). With kind permission from Elsevier: Ophthalmology, “Pericentral retinopathy and racial differences in hydroxychloroquine toxicity,” 2015, pp. 110–116, Melles and Marmor, Fig. 1

In Caucasian populations, hydroxychloroquine toxicity causes irreversible parafoveal photoreceptor loss with foveal sparing, resulting in paracentral scotomas. FAF shows a hyper-autofluorescent parafoveal ring corresponding to photoreceptor damage, which decreases in signal strength over time due to RPE atrophy (Fig. 14b) [180]. However, Asian populations tend to show a pericentral pattern of photoreceptor loss occurring well outside the parafoveal zone, with an odds ratio of 27.1 compared to the parafoveal pattern (Fig. 14d). Pericentral presentations tend to be diagnosed at higher cumulative dosages and at later stages of disease than parafoveal presentations, indicating that pericentral patterns are not as easily recognized on screening [181]. Late manifestations of the disease include bilateral bull’s eye maculopathy. If the drug regimen is not halted, the area of depigmentation may spread to involve the central fovea and even the entire fundus.

Screening guidelines for hydroxychloroquine toxicity by the American Academy of Ophthalmology recommend annual examinations starting at 1 year of use and annual evaluation with diagnostic testing including SD-OCT, perimetry, and mf-ERG [179, 182]. Compared to multi-focal ERG, FAF has a sensitivity of 73.7 % and is best used as a component of multi-modal imaging in the screening process [183, 184].

### Didanosine-induced retinal toxicity

**Fig. 15:**
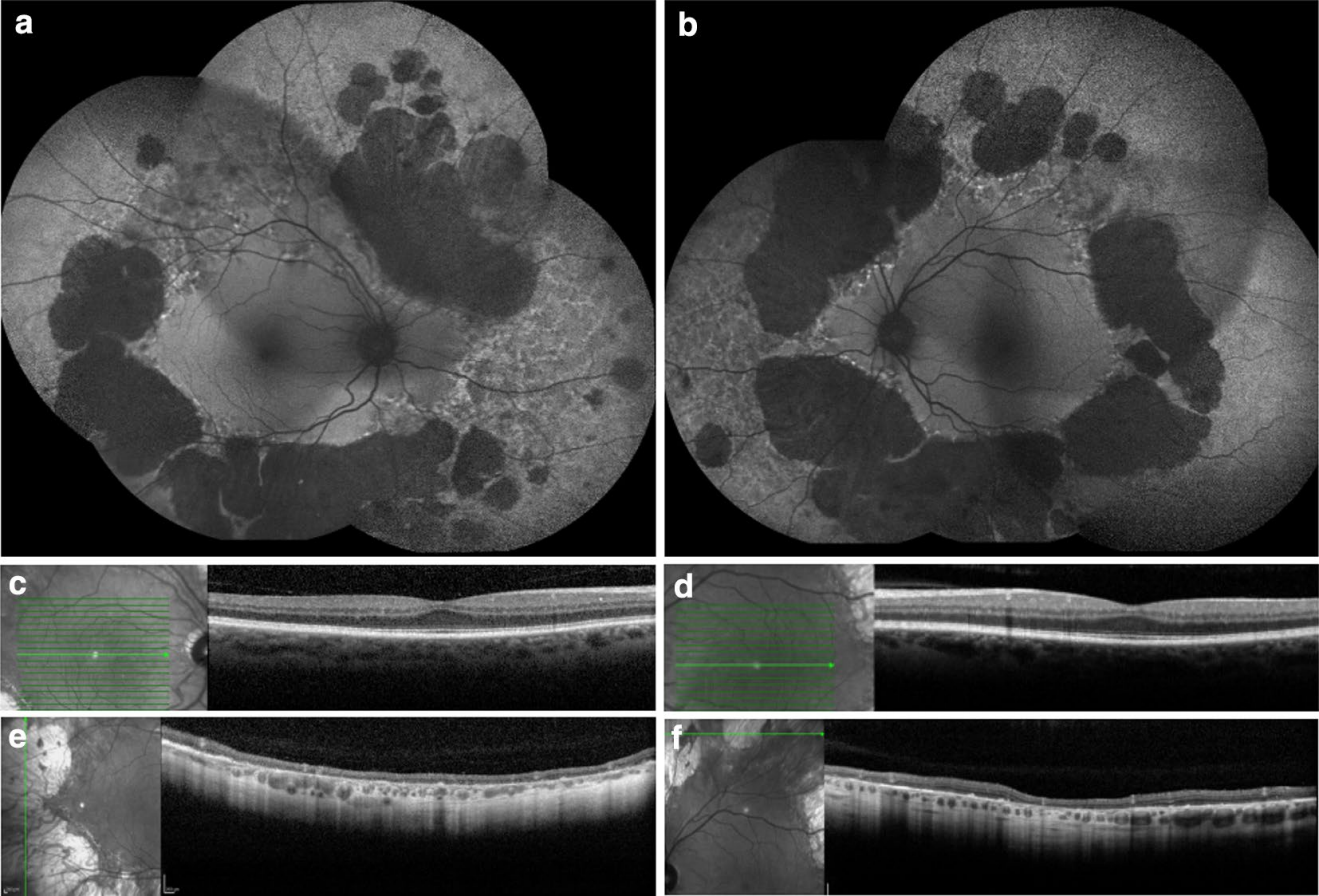
Multi-modal imaging of didanosine-induced (DDI) toxicity. Widefield FAF (**a**, **b**) demonstrated diffuse, bilateral midperipheral areas of well-circumscribed hypo-autofluoresence secondary to DDI toxicity. SD-OCT through the fovea (**c**, **d**) reveals macular sparing with intact retinal layers, while SD-OCT at the mid-periphery, through the lesion (**e**, **f**), demonstrates striking transmural atrophy with choroidal involvement. Reproduced with permission from [187]. Copyright© 2016 American Medical Association. All rights reserved

Didanosine (DDI) is a nucleoside reverse transcriptase inhibitor (NRTI) that was previously one of the mainstays for HIV treatment. However, this drug also inhibits the synthesis of mitochondrial DNA (mtDNA), resulting in mitochondrial toxicity and in rare cases precipitating a retinopathy similar to those seen in other mitochondrial disorders [185]. In the six cases described in the literature, adult DDI toxicity presents as sharply demarcated, midperipheral, concentric chorioretinal atrophy [186–189]. Widefield FAF is essential in making the diagnosis and shows a characteristic mottled hyper-autofluorescence and hypo-autofluorescence in the midperiphery that spares the central macula (Fig. 15) [187]. More advanced cases will show confluent midperipheral hypo-autofluorescent atrophy in each eye.

### Deferoxamine-induced retinal toxicity

As an iron chelator, deferoxamine is used to treat iron overload, such as in patients requiring chronic transfusions. Although the mechanism is not well understood, deferoxamine may injure the retina through direct toxic effects on RPE cells or chelation-induced iron, zinc, or copper deficiencies [190–192]. Deferoxamine retinopathy has various reported funduscopic manifestations, including pigmentary changes with RPE mottling, vitelliform lesions, and bull’s eye maculopathy [193–195].

FAF is more sensitive than ophthalmoscopy for detecting deferoxamine-induced retinal alterations and can be used to screen patients for toxicity and identify patients at high-risk for vision loss [196]. In a prospective case control study of 197 patients with β-thalassemia who received deferoxamine, 18 patients developed FAF abnormalities. Viola et al. characterized four distinct FAF patterns: minimal change, focal, patchy, and speckled patterns [196]. Patients with the minimal change pattern had the lowest incidence of vision deterioration, while patients with the patchy or speckled pattern had the most severe vision loss [196].

## Conclusion

Fundus autofluorescence provides information on the metabolic state and overall health of the RPE, and indirectly, the photoreceptor layer. Many imaging systems are available, each utilizing different optical properties that have distinct advantages and disadvantages. FAF is now an important tool for the evaluation of the prognosis and progression of geographic atrophy in AMD and for the phenotypic characterization of retinal dystrophies. FAF is also critical in the diagnosis of white dot syndromes and drug toxicities, among other entities. The applications of this simple, noninvasive imaging modality will continue to evolve and may replace more invasive procedures such as fluorescein angiography.

## Abbreviations

FAF: fundus autofluorescence
RPE: retinal pigment epithelium
AMD: age-related macular degeneration
cSLO: confocal scanning laser ophthalmoscopy
A2E: N-Retinyl-N-retinylidene ethanolamine
HRA: Heidelberg retinal angiograph
GA: geographic atrophy
PED: pigment epithelial detachment
GPS: fine granular with peripheral punctate spots
CSCR: central serous chorioretinopathy
AEPVM: acute exudative polymorphous vitelliform maculopathy
RP: retinitis pigmentosa
ERG: electroretinography
LCA: leber congenital amaurosis
MEWDS: multiple evanescent white dot syndrome
PIC: punctate inner choroidopathy
POHS: presumed ocular histoplasmosis syndrome
DDI: didanosine
NRTI: nucleoside reverse transcriptase inhibitor
mtDNA: mitochondrial DNA

## References

[1] Delori FC, Dorey CK, Staurenghi G, Arend O, Goger DG, Weiter JJ (1995). In vivo fluorescence of the ocular fundus exhibits retinal pigment epithelium lipofuscin characteristics. Invest Ophthalmol Vis Sci.

[2] Krebs I, Lois N, Forrester JV (2011). Fundus autofluorescence. Graefes Arch Clin Exp Ophthalmol.

[3] Wing GL, Blanchard GC, Weiter JJ (1978). The topography and age relationship of lipofuscin concentration in the retinal pigment epithelium. Invest Ophthalmol Vis Sci.

[4] Weiter JJ, Delori FC, Wing GL, Fitch KA (1986). Retinal pigment epithelial lipofuscin and melanin and choroidal melanin in human eyes. Invest Ophthalmol Vis Sci.

[5] Delori FC, Goger DG, Dorey CK (2001). Age-related accumulation and spatial distribution of lipofuscin in RPE of normal subjects. Invest Ophthalmol Vis Sci.

[6] Wu Y, Yanase E, Feng X, Siegel MM, Sparrow JR (2010). Structural characterization of bisretinoid A2E photocleavage products and implications for age-related macular degeneration. Proc Natl Acad Sci USA.

[7] Sparrow JR, Vollmer-Snarr HR, Zhou J (2003). A2E-epoxides damage DNA in retinal pigment epithelial cells. Vitamin E and other antioxidants inhibit A2E-epoxide formation. J Biol Chem.

[8] Ben-Shabat S, Itagaki Y, Jockusch S, Sparrow JR, Turro NJ, Nakanishi K (2002). Formation of a nonaoxirane from A2E, a lipofuscin fluorophore related to macular degeneration, and evidence of singlet oxygen involvement. Angew Chem.

[9] Feng J, Chen X, Sun X, Wang F, Sun X (2014). Expression of endoplasmic reticulum stress markers GRP78 and CHOP induced by oxidative stress in blue light-mediated damage of A2E-containing retinal pigment epithelium cells. Ophthalmic Res.

[10] Sparrow JR, Nakanishi K, Parish CA (2000). The lipofuscin fluorophore A2E mediates blue light-induced damage to retinal pigmented epithelial cells. Invest Ophthalmol Vis Sci.

[11] Lakkaraju A, Finnemann SC, Rodriguez-Boulan E (2007). The lipofuscin fluorophore A2E perturbs cholesterol metabolism in retinal pigment epithelial cells. Proc Natl Acad Sci USA.

[12] Sparrow JR, Cai B (2001). Blue light-induced apoptosis of A2E-containing RPE: involvement of caspase-3 and protection by Bcl-2. Invest Ophthalmol Vis Sci.

[13] Sparrow JR, Cai B, Jang YP, Zhou J, Nakanishi K (2006). A2E, a fluorophore of RPE lipofuscin, can destabilize membrane. Adv Exp Med Biol.

[14] Maeda A, Golczak M, Chen Y (2011). Primary amines protect against retinal degeneration in mouse models of retinopathies. Nat Chem Biol.

[15] Roberts JE, Kukielczak BM, Hu DN (2002). The role of A2E in prevention or enhancement of light damage in human retinal pigment epithelial cells. Photochem Photobiol.

[16] Crouch RK, Koutalos Y, Kono M, Schey K, Ablonczy Z (2015). A2E and Lipofuscin. Prog Mol Biol Transl Sci.

[17] Ablonczy Z, Higbee D, Anderson DM (2013). Lack of correlation between the spatial distribution of A2E and lipofuscin fluorescence in the human retinal pigment epithelium. Invest Ophthalmol Vis Sci.

[18] Smith RT, Bernstein PS, Curcio CA (2013). Rethinking A2E. Invest Ophthalmol Vis Sci.

[19] Freund KB, Laud K, Lima LH, Spaide RF, Zweifel S, Yannuzzi LA (2011). Acquired vitelliform lesions: correlation of clinical findings and multiple imaging analyses. Retina.

[20] Arnold JJ, Sarks JP, Killingsworth MC, Kettle EK, Sarks SH (2003). Adult vitelliform macular degeneration: a clinicopathological study. Eye (Lond).

[21] O’Gorman S, Flaherty WA, Fishman GA, Berson EL (1988). Histopathologic findings in Best’s vitelliform macular dystrophy. Arch Ophthalmol.

[22] Tso MO (1981). Pathology and pathogenesis of drusen of the optic nervehead. Ophthalmology.

[23] Sato T, Mrejen S, Spaide RF. Multimodal imaging of optic disc drusen. Am J Ophthalmol. 2013;156(2):275–82.e271. 10.1016/j.ajo.2013.03.039 23677136

[24] Van Schaik HJ, Alkemade C, Swart W, Van Best JA (1999). Autofluorescence of the diabetic and healthy human cornea in vivo at different excitation wavelengths. Exp Eye Res.

[25] Sparrow JM, Bron AJ, Brown NA, Neil HA (1992). Autofluorescence of the crystalline lens in early and late onset diabetes. Br J Ophthalmol.

[26] Abiko T, Abiko A, Ishiko S, Takeda M, Horiuchi S, Yoshida A (1999). Relationship between autofluorescence and advanced glycation end products in diabetic lenses. Exp Eye Res.

[27] Rovati L, Fankhauser F, Docchio F, Van Best J (1998). Diabetic retinopathy assessed by dynamic light scattering and corneal autofluorescence. J Biomed Opt.

[28] Sasamoto Y, Gomi F, Sawa M, Sakaguchi H, Tsujikawa M, Nishida K (2011). Effect of cataract in evaluation of macular pigment optical density by autofluorescence spectrometry. Invest Ophthalmol Vis Sci.

[29] Sharifzadeh M, Obana A, Gohto Y, Seto T, Gellermann W (2014). Autofluorescence imaging of macular pigment: influence and correction of ocular media opacities. J Biomed Opt.

[30] Sharifzadeh M, Bernstein PS, Gellermann W (2006). Nonmydriatic fluorescence-based quantitative imaging of human macular pigment distributions. J Opt Soc Am A Opt Image Sci Vis.

[31] Schweitzer D, Jentsch S, Schenke S, et al. Spectral and time-resolved studies on ocular structures. Paper presented at diagnostic optical spectroscopy in biomedicine IV; 2007/06/17, 2007; Munich.

[32] Spaide R, Holz FG, Schimtz-Valckenberg S, Spaide RF, Bird AC (2007). Imaging autofluorescence with a fundus camera. Atlas of fundus autofluorescence imaging.

[33] Feeney L (1978). Lipofuscin and melanin of human retinal pigment epithelium. Fluorescence, enzyme cytochemical, and ultrastructural studies. Invest Ophthalmol Vis Sci.

[34] Feeney-Burns L, Hilderbrand ES, Eldridge S (1984). Aging human RPE: morphometric analysis of macular, equatorial, and peripheral cells. Invest Ophthalmol Vis Sci.

[35] Keilhauer CN, Delori FC (2006). Near-infrared autofluorescence imaging of the fundus: visualization of ocular melanin. Invest Ophthalmol Vis Sci.

[36] Hu DN, Simon JD, Sarna T (2008). Role of ocular melanin in ophthalmic physiology and pathology. Photochem Photobiol.

[37] Burke JM, Kaczara P, Skumatz CM, Zareba M, Raciti MW, Sarna T (2011). Dynamic analyses reveal cytoprotection by RPE melanosomes against non-photic stress. Mol Vis.

[38] Wang Z, Dillon J, Gaillard ER (2006). Antioxidant properties of melanin in retinal pigment epithelial cells. Photochem Photobiol.

[39] Rozanowski B, Burke JM, Boulton ME, Sarna T, Rozanowska M (2008). Human RPE melanosomes protect from photosensitized and iron-mediated oxidation but become pro-oxidant in the presence of iron upon photodegradation. Invest Ophthalmol Vis Sci.

[40] Sarna T (1992). Properties and function of the ocular melanin—a photobiophysical view. J Photochem Photobiol, B.

[41] Sundelin SP, Nilsson SE, Brunk UT (2001). Lipofuscin-formation in cultured retinal pigment epithelial cells is related to their melanin content. Free Radic Biol Med.

[42] Sandberg MA, Gaudio AR, Miller S, Weiner A (1994). Iris pigmentation and extent of disease in patients with neovascular age-related macular degeneration. Invest Ophthalmol Vis Sci.

[43] Frank RN, Puklin JE, Stock C, Canter LA (2000). Race, iris color, and age-related macular degeneration. Trans Am Ophthalmol Soc.

[44] Nicolas CM, Robman LD, Tikellis G (2003). Iris colour, ethnic origin and progression of age-related macular degeneration. Clin Experiment Ophthalmol.

[45] Weiter JJ, Delori FC, Wing GL, Fitch KA (1985). Relationship of senile macular degeneration to ocular pigmentation. Am J Ophthalmol.

[46] Warrant EJ, Nilsson D-E (1998). Absorption of white light in photoreceptors. Vision Res.

[47] Prieto PM, McLellan JS, Burns SA (2005). Investigating the light absorption in a single pass through the photoreceptor layer by means of the lipofuscin fluorescence. Vision Res.

[48] Morgan JI, Pugh EN (2013). Scanning laser ophthalmoscope measurement of local fundus reflectance and autofluorescence changes arising from rhodopsin bleaching and regeneration. Invest Ophthalmol Vis Sci.

[49] Theelen T, Berendschot TT, Boon CJ, Hoyng CB, Klevering BJ (2008). Analysis of visual pigment by fundus autofluorescence. Exp Eye Res.

[50] Park SP, Siringo FS, Pensec N (2013). Comparison of fundus autofluorescence between fundus camera and confocal scanning laser ophthalmoscope-based systems. Ophthalmic Surg Lasers Imaging Retina.

[51] Spaide R (2008). Autofluorescence from the outer retina and subretinal space: hypothesis and review. Retina.

[52] Park SP, Siringo FS, Pensec N (2013). Comparison of fundus autofluorescence between fundus camera and confocal scanning laser ophthalmoscope-based systems. Ophthalmic Surgery Lasers Imaging Retina.

[53] Yamamoto M, Kohno T, Shiraki K (2009). Comparison of fundus autofluorescence of age-related macular degeneration between a fundus camera and a confocal scanning laser ophthalmoscope. Osaka City Med J.

[54] Lois N, Forrester JV (2016). Fundus autofluorescence.

[55] Deli A, Moetteli L, Ambresin A, Mantel I (2013). Comparison of fundus autofluorescence images acquired by the confocal scanning laser ophthalmoscope (488 nm excitation) and the modified Topcon fundus camera (580 nm excitation). Int Ophthalmol.

[56] Jorzik JJ, Bindewald A, Dithmar S, Holz FG (2005). Digital simultaneous fluorescein and indocyanine green angiography, autofluorescence, and red-free imaging with a solid-state laser-based confocal scanning laser ophthalmoscope. Retina.

[57] Sharp PF, Manivannan A, Xu H, Forrester JV (2004). The scanning laser ophthalmoscope—a review of its role in bioscience and medicine. Phys Med Biol.

[58] Trieschmann M, Spital G, Lommatzsch A (2003). Macular pigment: quantitative analysis on autofluorescence images. Graefes Arch Clin Exp Ophthalmol.

[59] Bellmann C, Rubin GS, Kabanarou SA, Bird AC, Fitzke FW (2003). Fundus autofluorescence imaging compared with different confocal scanning laser ophthalmoscopes. Br J Ophthalmol.

[60] Vingolo EM, Esposito M, Librando A, Huang Y-H, Salvatore S (2011). New retinal imaging for the visualization and analysis of vitreoretinal interface (VRI) by short-wavelength scanning laser ophthalmoscope (swSLO). Clin Ophthalmol.

[61] Acton JH, Cubbidge RP, King H, Galsworthy P, Gibson JM (2011). Drusen detection in retro-mode imaging by a scanning laser ophthalmoscope. Acta Ophthalmol.

[62] Diniz B, Ribeiro RM, Rodger DC, Maia M, Sadda S (2013). Drusen detection by confocal aperture-modulated infrared scanning laser ophthalmoscopy. Br J Ophthalmol.

[63] Ben Moussa N, Georges A, Capuano V, Merle B, Souied EH, Querques G (2015). MultiColor imaging in the evaluation of geographic atrophy due to age-related macular degeneration. Br J Ophthalmol.

[64] LaRocca F, Nankivil D, Farsiu S, Izatt JA (2014). True color scanning laser ophthalmoscopy and optical coherence tomography handheld probe. Biomed Opt Express.

[65] You QS, Bartsch DG, Espina M, Alam M, Camacho N, Mendoza N, Freeman WR (2015). Reproducibility of macular pigment optical density measurement by two-wavelength autofluorescence in a clinical setting. Retina.

[66] Weigert G, Kaya S, Pemp B (2011). Effects of lutein supplementation on macular pigment optical density and visual acuity in patients with age-related macular degeneration. Invest Ophthalmol Vis Sci.

[67] Bone RA, Landrum JT, Cains A (1992). Optical density spectra of the macular pigment in vivo and in vitro. Vision Res.

[68] Wustemeyer H, Moessner A, Jahn C, Wolf S (2003). Macular pigment density in healthy subjects quantified with a modified confocal scanning laser ophthalmoscope. Graefes Arch Clin Exp Ophthalmol.

[69] Delori FC, Goger DG, Hammond BR, Snodderly DM, Burns SA (2001). Macular pigment density measured by autofluorescence spectrometry: comparison with reflectometry and heterochromatic flicker photometry. J Opt Soc Am A Opt Image Sci Vis.

[70] Howells O, Eperjesi F, Bartlett H (2011). Measuring macular pigment optical density in vivo: a review of techniques. Graefes Arch Clin Exp Ophthalmol.

[71] Atkinson AMC. Imaged area of the retina. Accessed 21 Sept 2015.

[72] Witmer MT, Parlitsis G, Patel S, Kiss S (2013). Comparison of ultra-widefield fluorescein angiography with the Heidelberg Spectralis^®^ noncontact ultra-widefield module versus the Optos^®^ Optomap^®^. Clin Ophthalmol.

[73] Bonnay G, Nguyen F, Meunier I, Ducasse A, Hamel C, Arndt C (2011). Screening for retinal detachment using wide-field retinal imaging. J Fr Ophtalmol.

[74] Staurenghi G, Viola F, Mainster MA, Graham RD, Harrington PG (2005). Scanning laser ophthalmoscopy and angiography with a wide-field contact lens system. Arch Ophthalmol.

[75] Espina M, Barteselli G, Ma F (2014). Noncontact ultra-wide field lens system by Heidelberg spectralis. Invest Ophthalmol Vis Sci.

[76] Ben-Shabat S, Parish CA, Vollmer HR (2002). Biosynthetic studies of A2E, a major fluorophore of retinal pigment epithelial lipofuscin. J Biol Chem.

[77] Sparrow JR, Yoon KD, Wu Y, Yamamoto K (2010). Interpretations of fundus autofluorescence from studies of the bisretinoids of the retina. Invest Ophthalmol Vis Sci.

[78] Sparrow JR, Marsiglia M, Allikmets R (2015). Flecks in recessive stargardt disease: short-wavelength autofluorescence, near-infrared autofluorescence, and optical coherence tomography. Invest Ophthalmol Vis Sci.

[79] Rudolf M, Vogt SD, Curcio CA (2013). Histologic basis of variations in retinal pigment epithelium autofluorescence in eyes with geographic atrophy. Ophthalmology.

[80] Joseph A, Rahimy E, Freund KB, Sorenson JA, Sarraf D (2013). Fundus autofluorescence and photoreceptor bleaching in multiple evanescent white dot syndrome. Ophthalmic Surg Lasers Imaging Retina.

[81] Karadimas P, Paleokastritis GP, Bouzas EA (2006). Fundus autofluorescence imaging findings in retinal pigment epithelial tear. Eur J Ophthalmol.

[82] Klein R, Chou C, Klein BK, Zhang X, Meuer SM, Saaddine JB (2011). Prevalence of age-related macular degeneration in the us population. Arch Ophthalmol.

[83] Yonekawa Y, Miller JW, Kim IK (2015). Age-related macular degeneration: advances in management and diagnosis. J Clin Med.

[84] Warburton S, Davis WE, Southwick K (2007). Proteomic and phototoxic characterization of melanolipofuscin: correlation to disease and model for its origin. Mol Vis.

[85] Holz FG, Steinberg JS, Gobel A, Fleckenstein M, Schmitz-Valckenberg S (2015). Fundus autofluorescence imaging in dry AMD: 2014 Jules Gonin lecture of the Retina Research Foundation. Graefes Arch Clin Exp Ophthalmol.

[86] Bindewald A, Bird AC, Dandekar SS (2005). Classification of fundus autofluorescence patterns in early age-related macular disease. Invest Ophthalmol Vis Sci.

[87] Landa G, Rosen RB, Pilavas J, Garcia PM (2012). Drusen characteristics revealed by spectral-domain optical coherence tomography and their corresponding fundus autofluorescence appearance in dry age-related macular degeneration. Ophthalmic Res.

[88] Lois N, Owens SL, Coco R, Hopkins J, Fitzke FW, Bird AC (2002). Fundus autofluorescence in patients with age-related macular degeneration and high risk of visual loss. Am J Ophthalmol.

[89] Delori FC, Fleckner MR, Goger DG, Weiter JJ, Dorey CK (2000). Autofluorescence distribution associated with drusen in age-related macular degeneration. Invest Ophthalmol Vis Sci.

[90] Spaide RF, Curcio CA (2010). Drusen characterization with multimodal imaging. Retina.

[91] Mrejen S, Sarraf D, Mukkamala SK, Freund KB (2013). Multimodal imaging of pigment epithelial detachment: a guide to evaluation. Retina.

[92] Mimoun G, Soubrane G, Coscas G (1990). Macular drusen. J Fr Ophtalmol.

[93] Smith RT, Sohrab MA, Busuioc M, Barile G. Reticular macular disease. Am J Ophthalmol. 2009;148(5):733–43.e732. 10.1016/j.ajo.2009.06.028 PMC2786242 19878758

[94] Schmitz-Valckenberg S, Steinberg JS, Fleckenstein M, Visvalingam S, Brinkmann CK, Holz FG (2010). Combined confocal scanning laser ophthalmoscopy and spectral-domain optical coherence tomography imaging of reticular drusen associated with age-related macular degeneration. Ophthalmology.

[95] Pumariega NM, Smith RT, Sohrab MA, Letien V, Souied EH (2011). A prospective study of reticular macular disease. Ophthalmology.

[96] Pilotto E, Benetti E, Convento E (2013). Microperimetry, fundus autofluorescence, and retinal layer changes in progressing geographic atrophy. Can J Ophthalmol.

[97] Holz FG, Bindewald-Wittich A, Fleckenstein M, Dreyhaupt J, Scholl HP, Schmitz-Valckenberg S (2007). Progression of geographic atrophy and impact of fundus autofluorescence patterns in age-related macular degeneration. Am J Ophthalmol.

[98] Nagiel A, Freund KB, Jung JJ, Bhavsar K, Spaide RF, Sarraf D (2014). Origin and behavior of Type 3 neovascularization revealed by spectral-domain optical coherence tomography. Invest Ophthalmol Vis Sci.

[99] Vaclavik V, Vujosevic S, Dandekar SS, Bunce C, Peto T, Bird AC (2008). Autofluorescence imaging in age-related macular degeneration complicated by choroidal neovascularization: a prospective study. Ophthalmology.

[100] McBain VA, Townend J, Lois N (2007). Fundus autofluorescence in exudative age-related macular degeneration. Br J Ophthalmol.

[101] Camacho N, Barteselli G, Nezgoda JT (2015). Significance of the hyperautofluorescent ring associated with choroidal neovascularisation in eyes undergoing anti-VEGF therapy for wet age-related macular degeneration. Br J Ophthalmol.

[102] Batioglu F, Demirel S, Ozmert E, Oguz YG, Ozyol P (2014). Autofluorescence patterns as a predictive factor for neovascularization. Optom Vis Sci.

[103] Einbock W, Moessner A, Schnurrbusch UE, Holz FG, Wolf S (2005). Changes in fundus autofluorescence in patients with age-related maculopathy. Correlation to visual function: a prospective study. Graefes Arch Clin Exp Ophthalmol.

[104] Gass JD (1984). Pathogenesis of tears of the retinal pigment epithelium. Br J Ophthalmol.

[105] Sarraf D, Joseph A, Rahimy E (2014). Retinal pigment epithelial tears in the era of intravitreal pharmacotherapy: risk factors, pathogenesis, prognosis and treatment (an American Ophthalmological Society thesis). Trans Am Ophthalmol Soc.

[106] Nagiel A, Freund KB, Spaide RF, Munch IC, Larsen M, Sarraf D. Mechanism of retinal pigment epithelium tear formation following intravitreal anti-vascular endothelial growth factor therapy revealed by spectral-domain optical coherence tomography. Am J Ophthalmol. 2013;156(5):981–8.e982. 10.1016/j.ajo.2013.06.024 23972309

[107] Mendis R, Lois N (2014). Fundus autofluorescence in patients with retinal pigment epithelial (RPE) tears: an in vivo evaluation of RPE resurfacing. Graefes Arch Clin Exp Ophthalmol.

[108] Nagiel A, Sadda SR, Schwartz SD, Sarraf D (2015). Resolution of a giant pigment epithelial detachment with half-dose aflibercept. Retin Cases Brief Rep.

[109] Ferris FL, Wilkinson CP, Bird A (2013). Clinical classification of age-related macular degeneration. Ophthalmology.

[110] Saksens NTM, Fleckenstein M, Schmitz-Valckenberg S (2014). Macular dystrophies mimicking age-related macular degeneration. Prog Retin Eye Res.

[111] Teke MY, Elgin U, Nalcacioglu-Yuksekkaya P, Sen E, Ozdal P, Ozturk F (2014). Comparison of autofluorescence and optical coherence tomography findings in acute and chronic central serous chorioretinopathy. Int J Ophthalmol.

[112] Dinc UA, Tatlipinar S, Yenerel M, Gorgun E, Ciftci F (2011). Fundus autofluorescence in acute and chronic central serous chorioretinopathy. Clin Exp Optom.

[113] Iacono P, Battaglia PM, Papayannis A, La Spina C, Varano M, Bandello F (2015). Acute central serous chorioretinopathy: a correlation study between fundus autofluorescence and spectral-domain OCT. Graefes Arch Clin Exp Ophthalmol.

[114] Spaide RF, Klancnik JM (2005). Fundus autofluorescence and central serous chorioretinopathy. Ophthalmology.

[115] Fishkin N, Jang YP, Itagaki Y, Sparrow JR, Nakanishi K (2003). A2-rhodopsin: a new fluorophore isolated from photoreceptor outer segments. Org Biomol Chem.

[116] Zhang P, Wang H-Y, Zhang Z-F (2015). Fundus autofluorescence in central serous chorioretinopathy: association with spectral-domain optical coherence tomography and fluorescein angiography. Int J Ophthalmol.

[117] Shin JY, Choi HJ, Lee J, Choi M, Chung B, Byeon SH (2015). Fundus autofluorescence findings in central serous chorioretinopathy using two different confocal scanning laser ophthalmoscopes: correlation with functional and structural status. Graefes Arch Clin Exp Ophthalmol.

[118] Pang CE, Shah VP, Sarraf D, Freund KB. Ultra-widefield imaging with autofluorescence and indocyanine green angiography in central serous chorioretinopathy. Am J Ophthalmol. 2014;158(2):362–71.e362. 10.1016/j.ajo.2014.04.021 24794091

[119] Zhang K, Kniazeva M, Han M (2001). A 5-bp deletion in ELOVL4 is associated with two related forms of autosomal dominant macular dystrophy. Nat Genet.

[120] Lois N, Halfyard AS, Bird AC, Holder GE, Fitzke FW (2004). Fundus autofluorescence in Stargardt macular dystrophy-fundus flavimaculatus. Am J Ophthalmol.

[121] Cideciyan AV, Aleman TS, Swider M (2004). Mutations in ABCA4 result in accumulation of lipofuscin before slowing of the retinoid cycle: a reappraisal of the human disease sequence. Hum Mol Genet.

[122] Burke TR, Duncker T, Woods RL (2014). Quantitative fundus autofluorescence in recessive stargardt disease. Invest Ophthalmol Vis Sci.

[123] Boon CJ, Jeroen Klevering B, Keunen JE, Hoyng CB, Theelen T (2008). Fundus autofluorescence imaging of retinal dystrophies. Vision Res.

[124] Parodi MB, Iacono P, Campa C, Del Turco C, Bandello F (2014). Fundus autofluorescence patterns in Best vitelliform macular dystrophy. Am J Ophthalmol.

[125] Querques G, Zerbib J, Georges A (2014). Multimodal analysis of the progression of Best vitelliform macular dystrophy. Mol Vis.

[126] Bakall B, Radu RA, Stanton JB (2007). Enhanced accumulation of A2E in individuals homozygous or heterozygous for mutations in BEST1 (VMD2). Exp Eye Res.

[127] Wiklund A, Peebo BB (2013). Acute exudative polymorphous vitelliform maculopathy in a young woman: presymptomatic findings and 21-month follow-up. Retin Cases Brief Rep.

[128] Al-Dahmash SA, Shields CL, Bianciotto CG, Witkin AJ, Witkin SR, Shields JA (2012). Acute exudative paraneoplastic polymorphous vitelliform maculopathy in five cases. Ophthalmic Surg Lasers Imaging.

[129] Pang CE, Shields CL, Jumper JM, Yannuzzi LA. Paraneoplastic cloudy vitelliform submaculopathy in primary vitreoretinal lymphoma. Am J Ophthalmol. 2014;158(6):1253–61.e1252. 10.1016/j.ajo.2014.08.031 25174893

[130] Vianna RN, Muralha A, Muralha L (2003). Indocyanine-green angiography in acute idiopathic exudative polymorphous vitelliform maculopathy. Retina.

[131] Gass JD, Chuang EL, Granek H (1988). Acute exudative polymorphous vitelliform maculopathy. Trans Am Ophthalmol Soc.

[132] Grajewski RS, Schuler-Thurner B, Mauch C (2014). Ocular diseases in metastatic cutaneous melanoma: review of 108 consecutive patients in two German tertiary centers. Graefes Arch Clin Exp Ophthalmol.

[133] Vaclavik V, Ooi KG, Bird AC, Robson AG, Holder GE, Webster AR (2007). Autofluorescence findings in acute exudative polymorphous vitelliform maculopathy. Arch Ophthalmol.

[134] Gass JDM (1987). Stereoscopic atlas of macular diseases: diagnosis and treatment.

[135] Molday RS, Hicks D, Molday L (1987). Peripherin. A rim-specific membrane protein of rod outer segment discs. Invest Ophthalmol Vis Sci.

[136] Zhang K, Garibaldi DC, Li Y, Green WR, Zack DJ (2002). Butterfly-shaped pattern dystrophy: a genetic, clinical, and histopathological report. Arch Ophthalmol.

[137] Boon CJ, Klevering BJ, den Hollander AI (2007). Clinical and genetic heterogeneity in multifocal vitelliform dystrophy. Arch Ophthalmol.

[138] Chowers I, Tiosano L, Audo I, Grunin M, Boon CJ (2015). Adult-onset foveomacular vitelliform dystrophy: a fresh perspective. Prog Retin Eye Res.

[139] Renner AB, Tillack H, Kraus H (2004). Morphology and functional characteristics in adult vitelliform macular dystrophy. Retina.

[140] Duncker T, Greenberg JP, Ramachandran R (2014). Quantitative fundus autofluorescence and optical coherence tomography in best vitelliform macular dystrophy. Invest Ophthalmol Vis Sci.

[141] Furino C, Boscia F, Cardascia N, Sborgia L, Sborgia C (2008). Fundus autofluorescence, optical coherence tomography and visual acuity in adult-onset foveomacular dystrophy. Ophthalmologica.

[142] Parodi MB, Iacono P, Pedio M (2008). Autofluorescence in adult-onset foveomacular vitelliform dystrophy. Retina.

[143] Boon CJ, van Schooneveld MJ, den Hollander AI (2007). Mutations in the peripherin/RDS gene are an important cause of multifocal pattern dystrophy simulating STGD1/fundus flavimaculatus. Br J Ophthalmol.

[144] Mitamura Y, Mitamura-Aizawa S, Nagasawa T, Katome T, Eguchi H, Naito T (2012). Diagnostic imaging in patients with retinitis pigmentosa. J Med Invest.

[145] Ogura S, Yasukawa T, Kato A (2014). Wide-field fundus autofluorescence imaging to evaluate retinal function in patients with retinitis pigmentosa. Am J Ophthalmol.

[146] Murakami T, Akimoto M, Ooto S (2008). Association between abnormal autofluorescence and photoreceptor disorganization in retinitis pigmentosa. Am J Ophthalmol.

[147] Robson AG, Tufail A, Fitzke F (2011). Serial imaging and structure–function correlates of high-density rings of fundus autofluorescence in retinitis pigmentosa. Retina.

[148] Fleckenstein M, Charbel Issa P, Helb HM, Schmitz-Valckenberg S, Scholl HP, Holz FG (2008). Correlation of lines of increased autofluorescence in macular dystrophy and pigmented paravenous retinochoroidal atrophy by optical coherence tomography. Arch Ophthalmol.

[149] Fleckenstein M, Charbel Issa P, Fuchs HA (2009). Discrete arcs of increased fundus autofluorescence in retinal dystrophies and functional correlate on microperimetry. Eye (Lond).

[150] Greenstein VC, Duncker T, Holopigian K (2012). Structural and functional changes associated with normal and abnormal fundus autofluorescence in patients with retinitis pigmentosa. Retina.

[151] Robson AG, Egan CA, Luong VA, Bird AC, Holder GE, Fitzke FW (2004). Comparison of fundus autofluorescence with photopic and scotopic fine-matrix mapping in patients with retinitis pigmentosa and normal visual acuity. Invest Ophthalmol Vis Sci.

[152] Robson AG, Saihan Z, Jenkins SA (2006). Functional characterisation and serial imaging of abnormal fundus autofluorescence in patients with retinitis pigmentosa and normal visual acuity. Br J Ophthalmol.

[153] Popovic P, Jarc-Vidmar M, Hawlina M (2005). Abnormal fundus autofluorescence in relation to retinal function in patients with retinitis pigmentosa. Graefes Arch Clin Exp Ophthalmol.

[154] Seitz IP, Zhour A, Kohl S, Llavona P, Peter T, Wilhelm B, Zrenner E, Ueffing M, Bartz-Schmidt KU, Fischer MD (2015). Multimodal assessment of choroideremia patients defines pre-treatment characteristics. Graefes Arch Clin Exp Ophthalmol.

[155] Morgan JI, Han G, Klinman E (2014). High-resolution adaptive optics retinal imaging of cellular structure in choroideremia. Invest Ophthalmol Vis Sci.

[156] Pichi F, Morara M, Veronese C, Nucci P, Ciardella AP (2013). Multimodal imaging in hereditary retinal diseases. J Ophthalmol.

[157] Tolmachova T, Tolmachov O, Barnard A (2013). Functional expression of Rab escort protein 1 following AAV2-mediated gene delivery in the retina of choroideremia mice and human cells ex vivo. J Mol Med.

[158] Barnard AR, Groppe M, MacLaren RE (2015). Gene therapy for choroideremia using an adeno-associated viral (AAV) vector. Cold Spring Harb Perspect Med.

[159] Syed R, Sundquist SM, Ratnam K (2013). High-resolution images of retinal structure in patients with choroideremia. Invest Ophthalmol Vis Sci.

[160] Preising MN, Wegscheider E, Friedburg C, Poloschek CM, Wabbels BK, Lorenz B. Fundus autofluorescence in carriers of choroideremia and correlation with electrophysiologic and psychophysical data. Ophthalmology 2009;116(6):1201–9.e1201-1202. 10.1016/j.ophtha.2009.01.016 19376587

[161] Zeitz C, Robson AG, Audo I (2015). Congenital stationary night blindness: an analysis and update of genotype–phenotype correlations and pathogenic mechanisms. Prog Retin Eye Res.

[162] Yamamoto H, Simon A, Eriksson U, Harris E, Berson EL, Dryja TP (1999). Mutations in the gene encoding 11-cis retinol dehydrogenase cause delayed dark adaptation and fundus albipunctatus. Nat Genet.

[163] Schatz P, Preising M, Lorenz B (2010). Lack of autofluorescence in fundus albipunctatus associated with mutations in RDH5. Retina.

[164] Wang NK, Chuang LH, Lai CC (2012). Multimodal fundus imaging in fundus albipunctatus with RDH5 mutation: a newly identified compound heterozygous mutation and review of the literature. Doc Ophthalmol.

[165] Sergouniotis PI, Sohn EH, Li Z (2011). Phenotypic variability in RDH5 retinopathy (Fundus Albipunctatus). Ophthalmology.

[166] Genead MA, Fishman GA, Lindeman M (2010). Spectral-domain optical coherence tomography and fundus autofluorescence characteristics in patients with fundus albipunctatus and retinitis punctata albescens. Ophthalmic Genet.

[167] Moiseyev G, Chen Y, Takahashi Y, Wu BX, Ma JX (2005). RPE65 is the isomerohydrolase in the retinoid visual cycle. Proc Natl Acad Sci USA.

[168] Schatz P, Preising M, Lorenz B, Sander B, Larsen M, Rosenberg T (2011). Fundus albipunctatus associated with compound heterozygous mutations in RPE65. Ophthalmology.

[169] Crawford CM, Igboeli O (2013). A review of the inflammatory chorioretinopathies: the white dot syndromes. ISRN Inflamm.

[170] Marsiglia M, Gallego-Pinazo R, Cunha de Souza E, Munk MR, Yu S, Mrejen S, Cunningham ET, Lujan BJ, Goldberg NR, Albini TA, Gaudric A, Francais C, Rosen RB, Freund KB, Jampol LM, Yannuzzi LA (2016). Expanded clinical spectrum of multiple evanescent white dot syndrome with multimodal imaging. Retina.

[171] Li M, Zhang X, Wen F (2015). The fundus autofluorescence spectrum of punctate inner choroidopathy. J Ophthalmol.

[172] Yenerel NM, Kucumen B, Gorgun E, Dinc UA (2008). Atypical presentation of multiple evanescent white dot syndrome (MEWDS). Ocul Immunol Inflamm.

[173] Furino C, Boscia F, Cardascia N, Alessio G, Sborgia C (2009). Fundus autofluorescence and multiple evanescent white dot syndrome. Retina.

[174] Essex RW, Wong J, Fraser-Bell S (2010). Punctate inner choroidopathy: clinical features and outcomes. Arch Ophthalmol.

[175] Spaide RF, Goldberg N, Freund KB (2013). Redefining multifocal choroiditis and panuveitis and punctate inner choroidopathy through multimodal imaging. Retina.

[176] Klufas MA, O’Hearn T, Sarraf D (2015). Optical coherence tomography angiography and widefield fundus autofluorescence in punctate inner choroidopathy. Retin Cases Brief Rep.

[177] Riaz KM, Jampol LM, Mirza RG (2012). Fundus autofluorescence imaging in punctate inner choroidopathy with blind spot enlargement. Ocul Immunol Inflamm.

[178] Wolfe F, Marmor MF (2010). Rates and predictors of hydroxychloroquine retinal toxicity in patients with rheumatoid arthritis and systemic lupus erythematosus. Arthritis Care Res.

[179] Marmor MF, Kellner U, Lai TY, Lyons JS, Mieler WF (2011). Revised recommendations on screening for chloroquine and hydroxychloroquine retinopathy. Ophthalmology.

[180] Kellner U, Renner AB, Tillack H (2006). Fundus autofluorescence and mfERG for early detection of retinal alterations in patients using chloroquine/hydroxychloroquine. Invest Ophthalmol Vis Sci.

[181] Melles RB, Marmor MF (2015). Pericentral retinopathy and racial differences in hydroxychloroquine toxicity. Ophthalmology.

[182] Elder M, Rahman AM, McLay J (2006). Early paracentral visual field loss in patients taking hydroxychloroquine. Arch Ophthalmol.

[183] Cukras C, Huynh N, Vitale S, Wong WT, Ferris FL, Sieving PA (2015). Subjective and objective screening tests for hydroxychloroquine toxicity. Ophthalmology.

[184] Gorovoy IR, Gorovoy MS (2013). Fundus autofluorescence is not the best early screen for hydroxychloroquine toxicity—reply. JAMA Ophthalmol.

[185] Wang H, Lemire BD, Cass CE (1996). Zidovudine and dideoxynucleosides deplete wild-type mitochondrial DNA levels and increase deleted mitochondrial DNA levels in cultured Kearns–Sayre syndrome fibroblasts. Biochim Biophys Acta.

[186] Pinto R, Lino S, Nogueira V, Fonseca A, Ornelas C (2013). A woman with didanosine retinopathy and non-cirrhotic portal hypertension. Int J STD AIDS.

[187] Gabrielian A, MacCumber MM, Kukuyev A, Mitsuyasu R, Holland GN, Sarraf D (2013). Didanosine-associated retinal toxicity in adults infected with human immunodeficiency virus. JAMA Ophthalmol.

[188] Whitcup SM, Dastgheib K, Nussenblatt RB, Walton RC, Pizzo PA, Chan CC (1994). A clinicopathologic report of the retinal lesions associated with didanosine. Arch Ophthalmol.

[189] Cobo J, Ruiz MF, Figueroa MS (1996). Retinal toxicity associated with didanosine in HIV-infected adults. AIDS.

[190] De Virgiliis S, Congia M, Turco MP (1988). Depletion of trace elements and acute ocular toxicity induced by desferrioxamine in patients with thalassaemia. Arch Dis Child.

[191] Klettner A, Koinzer S, Waetzig V, Herdegen T, Roider J (2010). Deferoxamine mesylate is toxic for retinal pigment epithelium cells in vitro, and its toxicity is mediated by p38. Cutan Ocul Toxicol.

[192] Chaston TB, Richardson DR (2003). Iron chelators for the treatment of iron overload disease: relationship between structure, redox activity, and toxicity. Am J Hematol.

[193] Bansal V, Elgarbly I, Ghanchi FD, Atkinson PL (2003). Bull’s eye maculopathy with deferoxamine. Eur J Haematol.

[194] Haimovici R, D’Amico DJ, Gragoudas ES, Sokol S (2002). The expanded clinical spectrum of deferoxamine retinopathy. Ophthalmology.

[195] Genead MA, Fishman GA, Anastasakis A, Lindeman M (2010). Macular vitelliform lesion in desferrioxamine-related retinopathy. Doc Ophthalmol.

[196] Viola F, Barteselli G, Dell’arti L (2012). Abnormal fundus autofluorescence results of patients in long-term treatment with deferoxamine. Ophthalmology.

